# A Human Trypanosome Suppresses CD8^+^ T Cell Priming by Dendritic Cells through the Induction of Immune Regulatory CD4^+^ Foxp3^+^ T Cells

**DOI:** 10.1371/journal.ppat.1005698

**Published:** 2016-06-22

**Authors:** Jonatan Ersching, Alexandre Salgado Basso, Vera Lucia Garcia Kalich, Karina Ramalho Bortoluci, Maurício M. Rodrigues

**Affiliations:** 1 Centro de Terapia Celular e Molecular (CTCMol), Universidade Federal de São Paulo-Escola Paulista de Medicina, São Paulo, São Paulo, Brazil; 2 Departamento de Microbiologia, Imunologia e Parasitologia, Universidade Federal de São Paulo-Escola Paulista de Medicina, São Paulo, São Paulo, Brazil; 3 Departamento de Imunologia, Instituto de Ciências Biomédicas, Universidade de São Paulo, São Paulo, São Paulo, Brazil; 4 Departamento de Ciências Biológicas, Universidade Federal de São Paulo, São Paulo, São Paulo, Brazil; University of Texas Medical Branch, UNITED STATES

## Abstract

Although CD4^+^ Foxp3^+^ T cells are largely described in the regulation of CD4^+^ T cell responses, their role in the suppression of CD8^+^ T cell priming is much less clear. Because the induction of CD8^+^ T cells during experimental infection with *Trypanosoma cruzi* is remarkably delayed and suboptimal, we raised the hypothesis that this protozoan parasite actively induces the regulation of CD8^+^ T cell priming. Using an *in vivo* assay that eliminated multiple variables associated with antigen processing and dendritic cell activation, we found that injection of bone marrow-derived dendritic cells exposed to *T*. *cruzi* induced regulatory CD4^+^ Foxp3^+^ T cells that suppressed the priming of transgenic CD8^+^ T cells by peptide-loaded BMDC. This newly described suppressive effect on CD8^+^ T cell priming was independent of IL-10, but partially dependent on CTLA-4 and TGF-β. Accordingly, depletion of Foxp3^+^ cells in mice infected with *T*. *cruzi* enhanced the response of epitope-specific CD8^+^ T cells. Altogether, our data uncover a mechanism by which *T*. *cruzi* suppresses CD8^+^ T cell responses, an event related to the establishment of chronic infections.

## Author Summary

CD8^+^ T lymphocytes mediate immunity to intracellular pathogens by killing infected cells. However, some pathogens are able to evade the response of CD8^+^ T cells and, thus, establish chronic infections. This is the case of *Trypanosoma cruzi*, the protozoan parasite that causes Chagas disease. Here, we investigated the basis of the suboptimal response of CD8^+^ T cells during *T*. *cruzi* infection. We observed that cells incubated with the parasite and then adoptively transferred into mice are able to convert an optimal *in vivo* response of transgenic CD8^+^ T cells specific to an unrelated epitope into suboptimal. The mechanism of this disturbance relies on the induction of regulatory CD4^+^ Foxp3^+^ T cells that interfere with the priming of CD8^+^ T cells by dendritic cells. These findings illustrate the involvement of regulatory T cells in the regulation of CD8^+^ T cell priming and contribute to understand how *T*. *cruzi* evades host immunity to establish a chronic infection.

## Introduction

Mouse models of self-curing infections with lymphocytic choriomeningitis virus (LCMV) and *Listeria monocytogenes* enable CD8^+^ T cells to be rapidly activated, proliferate and peak between 5 to 10 days post-infection. These lymphocytes differentiate into effector cells and participate in pathogen control and clearance [1–6]. Conversely, during experimental mouse infections with *Mycobacterium tuberculosis*, *Salmonella spp*, *Toxoplasma gondii* or *Trypanosoma cruzi*, the peak of the primary CD8^+^ T cell immune response occurs only later than 20 days following challenge, in association with host death or the establishment of chronic infections [7–13]. Understanding the mechanisms underlying the delayed onset of CD8^+^ T cell responses in these cases entail the development of interventions to restrain infection. However, such mechanisms remain ill defined.

*Trypanosoma cruzi* is an intracellular protozoan that currently infects more than 10 million people in the Americas and may cause a chronic digestive and/or cardiac pathology known as Chagas disease. Murine models of infection revealed that CD8^+^ T cells are essential for *T*. *cruzi* control [10–12,14,15]. However, the primary response of specific CD8^+^ T cells after *T*. *cruzi* infection is significantly delayed and marked by a high frequency of proapoptotic cells [10,12,14–16]. On the other hand, coopting viruses as genetic vectors to induce faster and long-lasting CD8^+^ T cell responses against *T*. *cruzi* has been shown feasible in either prophylactic or therapeutic vaccination protocols [16–18].

Here, we tested the hypothesis that this contrasting control of the onset of CD8^+^ T cell immunity induced by *T*. *cruzi* infection as compared to genetic immunization with viral vectors occurs very early during the priming of CD8^+^ T cells by dendritic cells (DC) and involves active mechanisms of suppression. In order to precisely identify these mechanisms and eliminate other variables related to antigen uptake, processing and presentation, we employed a simple and controlled system in which we used *in vitro* generated bone marrow-derived dendritic cells (BMDC) stimulated with LPS and loaded with the ovalbumin MHC I-restricted epitope SIINFEKL (BMDC-SIINFEKL) to optimally prime cognate OTI transgenic CD8^+^ T cells *in vivo*. By using this system we normalized the provision of signals required for CD8^+^ T cell activation and were able to study the specific impact of *T*. *cruzi* in an otherwise maximized response.

We observed that *T*. *cruzi*-exposed BMDC-SIINFEKL induced regulatory CD4^+^ Foxp3^+^ T cells that inhibited proliferation, differentiation and cytokine production of OTI CD8^+^ T cells. Furthermore, this newly described suppression was shown to be independent of IL-10 and partially mediated by CTLA-4 and TGF-β.

## Results

### Suboptimal *in vivo* priming of CD8^+^ T cells by *T*. *cruzi*-exposed DC

**Fig 1:**
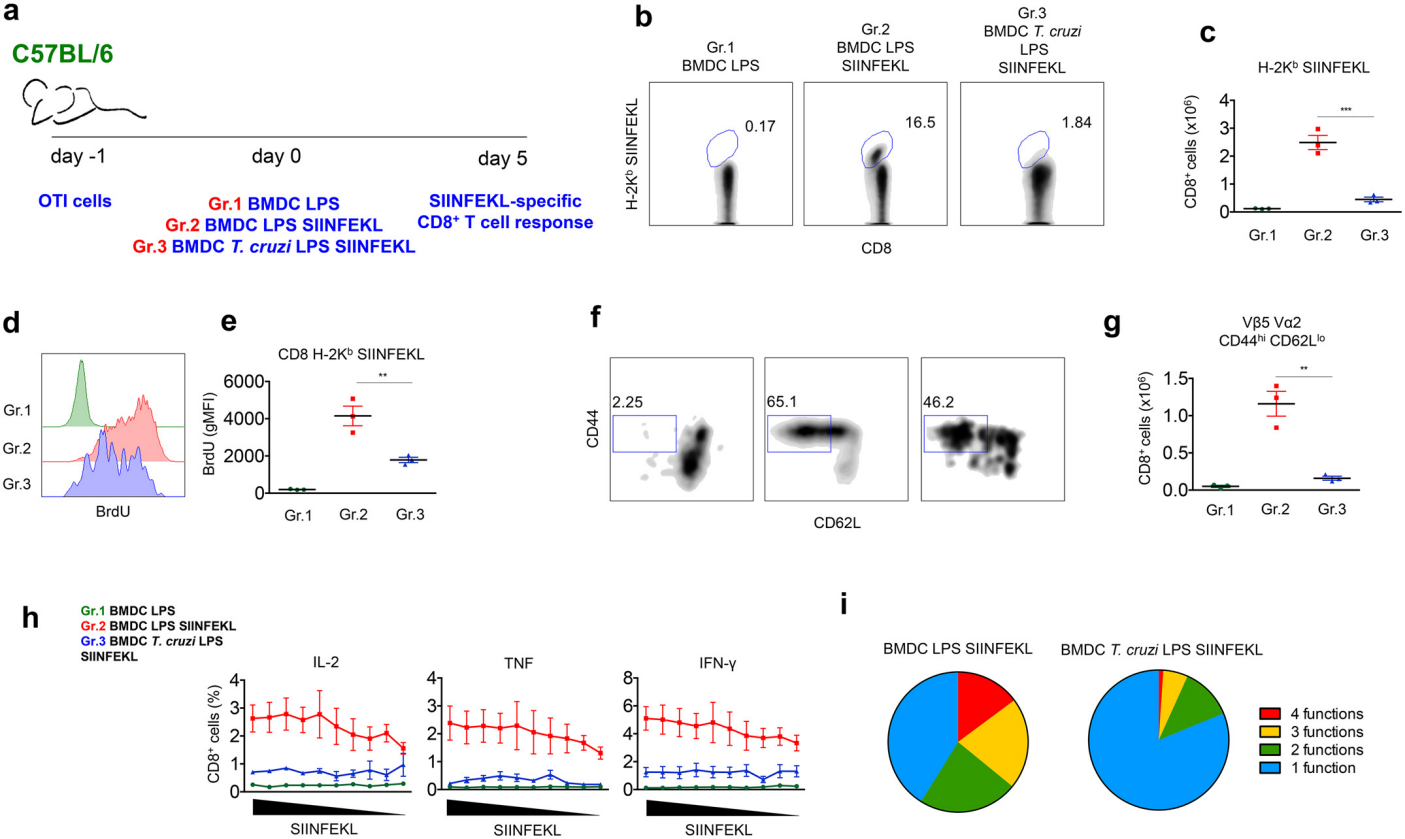
Suboptimal expansion and differentiation of OTI CD8^+^ T cells upon *in vivo* stimulation with *T*. *cruzi*-exposed BMDC-SIINFEKL. a- 1 x 10^4^ OTI cells were adoptively transferred into C57BL/6 mice prior to transfer of 5 x 10^5^ control BMDC exposed to LPS only (Gr.1) or 5 x 10^5^ BMDC exposed to LPS and loaded with SIINFEKL peptide (Gr. 2) or 5 x 10^5^ BMDC previously exposed to *T*. *cruzi* and LPS and loaded with SIINFEKL peptide (Gr. 3). The SIINFEKL-specific immune response was assessed after 5 days. b and c- The numbers of SIINFEKL-specific CD8^+^ T cells were determined by H-2K^b^ SIINFEKL tetramer staining. d and e- Proliferation of OTI cells was measured by BrdU incorporation in tetramer-positive CD8^+^ T cells. f and g- The ability of naïve OTI cells to differentiate into effector cells was evaluated by CD44 and CD62L staining of TCR Vα2 Vβ5 double positive CD8 cells. h—After 5 days, spleen cells were harvested and restimulated *ex vivo* with SIINFEKL peptide (ranging from 1x10^-6^ M to 3X10^-11^ M). The numbers of IL-2 and/or TNF and/or IFN-γ-producing CD8^+^ T cells were assessed by intracellular staining. i—Proportion of polyfunctional CD8^+^ T cells stained for CD107a and/or one, two or three cytokines (IL-2, IFN-γ and TNF) combined after restimulation with SIINFEKL peptide (1x10^-6^ M). Results are one of three separate experiments expressed as individual values and the mean ± SEM of each group. Asterisks indicate significant differences between groups (**P<0.01, ***P<0.001, One-way ANOVA followed by Tukey post-hoc test).

To investigate whether BMDC exposure to *T*. *cruzi* could affect their ability to prime specific CD8^+^ T cells, we set up an experimental model using the peptide SIINFEKL (MHC I-restricted epitope from ovalbumin) as antigen and cognate transgenic CD8^+^ T cells (OTI cells) as responder cells. Transgenic OTI cells harboring Vα2 Vβ5 TCR specific for SIINFEKL were transferred into naïve C57BL/6 mice. One day later, animals were transferred with BMDC previously stimulated with LPS and loaded or not with SIINFEKL peptide. Alternatively, BMDC were *in vitro-*exposed to *T*. *cruzi* 24 h before LPS stimulation and SIINFEKL peptide loading. Five days after transfer, the specific response of OTI cells was evaluated in the spleen, as depicted in Fig 1a.

In comparison to baseline levels observed in control mice that received BMDC stimulated only with LPS (Gr. 1), the transfer of BMDC-SIINFEKL (Gr. 2) induced the expansion of OTI CD8^+^ T cells, as measured by H-2K^b^ SIINFEKL tetramer staining (Fig 1b and 1c) and Vα2 and Vβ5 TCR staining (S1a and S1b Fig). This increase was due to the proliferation of OTI cells, as demonstrated by BrdU incorporation in tetramer-positive CD8^+^ T cells (Fig 1d and 1e). We also observed that Vα2^+^ Vβ5^+^ CD8^+^ cells differentiated into an effector phenotype upon stimulation with BMDC-SIINFEKL, as indicated by CD44 upregulation and CD62L downregulation (Fig 1f and 1g). In remarkable contrast to the scenario described above, the response of OTI cells from mice injected with *T*. *cruzi*-exposed BMDC-SIINFEKL (Gr. 3) was significantly impaired, as indicated by their numbers, proliferation and differentiation into an effector phenotype (Fig 1b–1g).

By intracellular staining (ICS), we also observed that 5 days after BMDC transfer, the frequencies of splenic CD8^+^ T cells positive for IL-2, TNF or IFN-γ upon *ex-vivo* restimulation with different concentrations of SIINFEKL peptide were significantly lower in mice injected with *T*. *cruzi*-exposed BMDC-SIINFEKL as compared to animals that received BMDC-SIINFEKL (Fig 1h). Not only the quantity, but also the quality of the cytokine response was altered when BMDC-SIINFEKL were previously exposed to *T*. *cruzi*. Polyfunctional cells, defined as simultaneously positive for at least two parameters (IL-2 and/or TNF and/or IFN-γ and/or the degranulation marker CD107a), were dominant among the total cytokine-producing CD8^+^ T cells from animals that received BMDC-SIINFEKL, whereas single-positive cells prevailed when animals were injected with *T*. *cruzi*-exposed BMDC-SIINFEKL (Fig 1i). Production of IL-4, IL-10 or IL-17 was below ICS detection limits in CD8^+^ T cells restimulated with SIINFEKL from animals of all groups. Elispot to detect IFN-γ producing cells after SIINFEKL restimulation confirmed an impaired cytokine response of OTI cells from animals injected with *T*. *cruzi*-exposed BMDC-SIINFEKL when compared to BMDC-SIINFEKL (S1c and S1d Fig).

A possible caveat in the experiments described above could be that H-2K^b^-restricted CD8^+^ T cells specific for *T*. *cruzi* epitopes could compete with the transgenic OTI CD8^+^ T cells for priming by the same BMDC. However, on day 5 after *T*. *cruzi*-exposed BMDC-SIINFEKL transfer, no IFN-γ and/or TNF cytokine responses of splenic CD8^+^ T cells was observed upon *ex vivo* restimulation with the peptides VNHRFTLV and ANYKFTLV, which correspond to the two *T*. *cruzi* immunodominant H-2K^b^-restricted epitopes (S2 Fig). This observation ruled out the possibility that the lower response of OTI cells in mice from Gr.3 was due to competition with dominating parasite-specific CD8^+^ T lymphocytes. In fact, the CD8^+^ T cell immune responses to the peptides VNHRFTLV and ANYKFTLV could be measured only later (after 12 days), as we have previously described [12,15] (S2 Fig).

In addition, when BMDC were exposed to adenovirus type 5 expressing amastigote surface protein 2 from *T*. *cruzi* prior to LPS stimulation and SIINFEKL load, the response of OTI CD8^+^ T cells was not impaired (S3 Fig). From these data, we concluded that exposure to *T*. *cruzi* significantly disturbs DC ability to induce cognate naïve OTI CD8^+^ T cells to proliferate, differentiate and produce cytokines *in vivo*.

### DC exposed to *T*. *cruzi* are functionally matured and actively induce the suppression of CD8^+^ T cell priming

**Fig 2:**
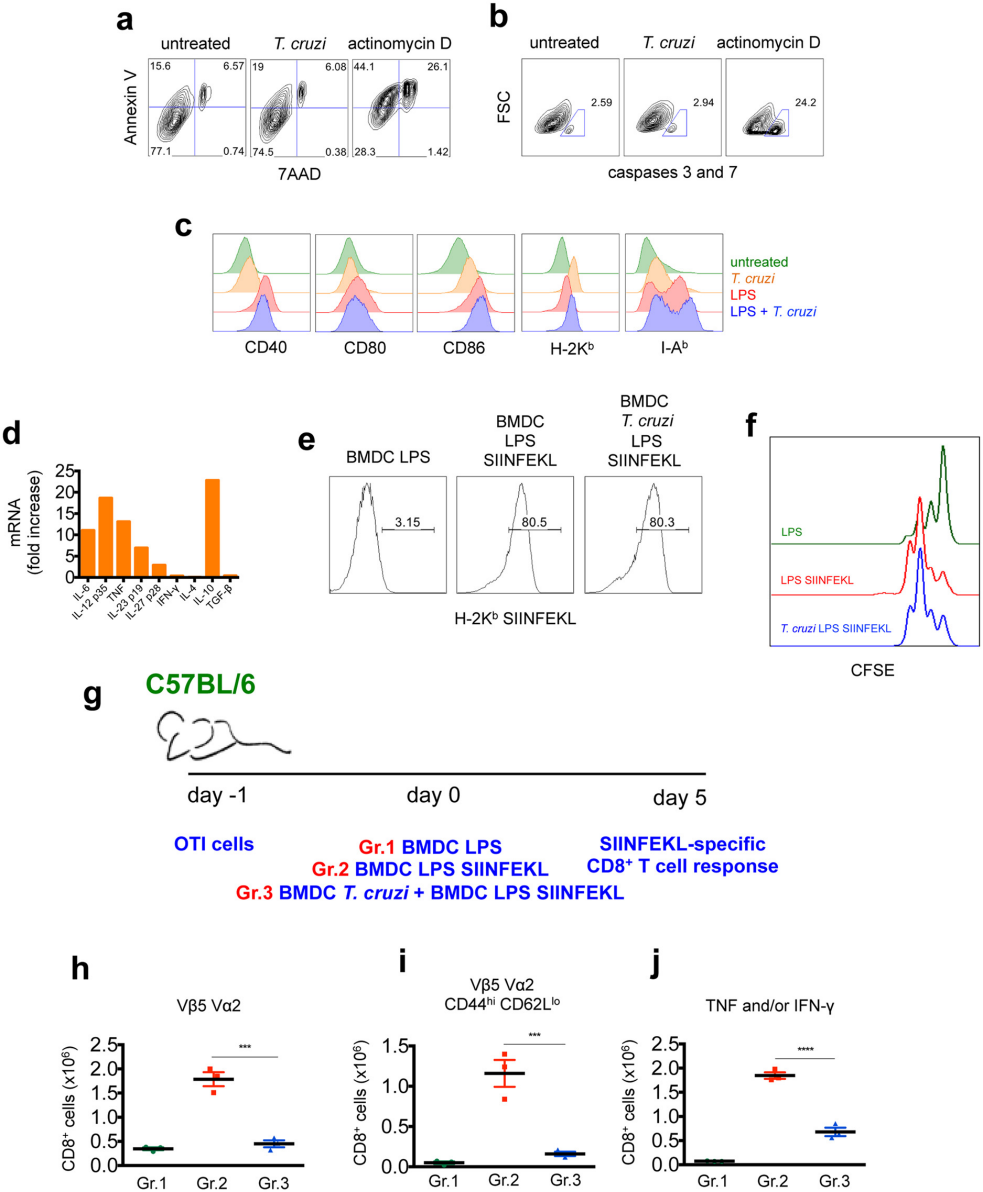
BMDC exposed to *T*. *cruzi* are fully matured and actively suppress CD8^+^ T cell priming *in trans*. a and b- To assess viability, BMDC were left untreated or exposed to *T*. *cruzi* or actinomycin D prior to staining with Annexin V and 7AAD or probes to detect the active forms of caspase 3 and 7 (gated on CD11c^+^ cells). c and d- The upregulation of MHC and co-stimulatory molecules on the surface of LPS and/or *T*. *cruzi-*exposed BMDC was assessed by flow cytometry (histograms were gated in CD11c^+^ cells) and the cytokine response was assessed by RT-PCR. e- BMDC were incubated with *T*. *cruzi*, stimulated with LPS, loaded with SIINFEKL peptide and the complex H-2K^b^-SIINFEKL on the surface of CD11c^+^ cells was stained with 25-D1.16 antibody. f- The ability of naïve OTI CD8^+^ T cells to proliferate *in vitro* was assessed by CFSE dilution after 3 days of co-culture with the indicated BMDC loaded with SIINFEKL peptide and exposed or not to *T*. *cruzi*. g- 1 x 10^4^ OTI cells were adoptively transferred into C57BL/6 mice before the transfer of 5 x 10^5^ BMDC (Gr.1), 5 x 10^5^ BMDC-SIINFEKL (Gr.2) or 5 x 10^5^ BMDC-SIINFEKL and 5 x 10^5^ *T*. *cruzi*-exposed BMDC (Gr.3). The SIINFEKL-specific immune response was assessed after 5 days. h- Numbers of SIINFEKL-specific CD8^+^ T cells were determined by TCR Vα2 Vβ5 staining. i- The ability of naïve OTI cells to differentiate into effector cells was evaluated by CD44 and CD62L staining of TCR Vα2^+^ Vβ5^+^ CD8^+^ T cells. j- Spleen cells were restimulated *ex vivo* with SIINFEKL peptide and numbers of TNF and/or IFN-γ-producing CD8^+^ T cells were determined by ICS. Results are one of three separate experiments expressed as individual values and the mean ± SEM of each group. Asterisks represent significant difference between the indicated groups (***P<0.001, ****P<0.0001 One-way ANOVA followed by Tukey post-hoc test).

To investigate whether *T*. *cruzi* could interfere with the activation and antigen presentation function of DC, we generated BMDC and exposed them *in vitro* to *T*. *cruzi*. After 24 h of exposure to the parasite, approximately 60% of BMDC were infected and featured intracellular amastigotes detected by Giemsa-staining. BMDC were stained with 7AAD, annexin-V, and fluorescent probes to detect active forms of caspases 3 and 7, and analyzed by flow cytometry. As shown in Fig 2a and 2b, *T*. *cruzi*-exposed BMDC and untreated cells were equally viable, whereas control cells treated with actinomycin D showed a significant degree of apoptosis.

To evaluate the ability of BMDC to modulate expression of MHC and co-stimulatory molecules, BDMC were incubated with *T*. *cruzi* for 24 h in the absence or presence of LPS and then FACS-stained for CD40, CD80, CD86, H-2K^b^ and I-A^b^. As shown in Fig 2c, *T*. *cruzi* alone induced the upregulation of MHC and co-stimulatory molecules on BMDC. Furthermore, the upregulation achieved by LPS exposure was not inhibited by the parasite.

BMDC gene expression of cytokines in response to *T*. *cruzi* was assessed by RT-PCR (Fig 2d). Of note, presence of the parasite induced mRNA expression of cytokines important for the induction of CD8^+^ T cell responses, including IL-6, IL-12, TNF, IL-23, and IL-27, concomitantly with the immune suppressive cytokine IL-10. When BMDC were exposed to LPS, gene expression of these cytokines was highly upregulated, regardless of *T*. *cruzi* pre-exposure, suggesting that cytokine levels induced in BMDC LPS SIINFEKL, even in presence of the parasite, could be enough to induce the priming of CD8^+^ T cells. However, LPS alone was more potent at triggering this upregulation than *T*. *cruzi* and LPS combined (S4a Fig).

To explain the suboptimal *in vivo* induction of OTI CD8^+^ T cells by *T*. cruzi-exposed BMDC-SIINFEKL, we reasoned that H-2K^b^-SIINFEKL peptide complexes could be either not formed or removed from the surface of *T*. *cruzi*-exposed BMDC-SIINFEKL. To quantify the amount of cognate peptide provided, we surface-stained BMDC with the 25-D1.16 antibody, which recognizes the H2K^b^-SIINFEKL complexes. As depicted in Fig 2e, BMDC-SIINFEKL efficiently displayed the complex H-2K^b^-SIINFEKL regardless of *T*. *cruzi* pre-exposure. Additionally, BMDC-SIINFEKL previously exposed or not to *T*. *cruzi* were equally able to induce proliferation of naïve OTI CD8^+^ T cells *in vitro*, as measured by CFSE dilution after three days in co-culture (Fig 2f). Accordingly, naïve OTI CD8^+^ T cells equally secreted IFN-γ *in vitro* after five days in co-culture with BMDC SIINFEKL or *T*. cruzi-exposed BMDC SIINFEKL (S4b Fig).

Altogether, these data indicate that BMDC exposed to *T*. *cruzi* are able to provide the three signals required for T cell activation: i) MHCI-peptide complex, ii) co-stimulatory molecules, and iii) inflammatory cytokines.

To better understand the mechanisms responsible for the disturbance of the CD8^+^ T cell response caused by the exposure of DC to *T*. *cruzi*, we evaluated the *in vivo* activation of OTI CD8^+^ T cells upon transfer of BMDC-SIINFEKL (carrying the cognate peptide) and *T*. *cruzi*-exposed BMDC (not carrying the cognate peptide) simultaneously into the same mice (outlined in Fig 2g). Under these conditions, if *T*. *cruzi* impairs the antigen presentation function of BMDC loaded with SIINFEKL peptide, one would expect that separating the cells that carry the peptide (transferred i.v. in the right retro-orbital sinus) from the cells exposed to the parasite (transferred i.v. in the left retro-orbital sinus) could restore the stimulation of OTI CD8^+^ T cells to optimal levels. However, we observed that the proliferation (Fig 2h), differentiation into effector phenotype (CD44^hi^ CD62L^low^) (Fig 2i) and cytokine response (Fig 2j) of OTI cells were significantly impaired in mice that received both, parasite-exposed BMDC and BMDC-SIINFEKL, compared to the animals that received only BMDC-SIINFEKL.

It is important to note that, exactly as reported for BMDC, transfer of splenic DC exposed to *T*. *cruzi* also led to the *in trans* impairment of the response of OTI CD8+ T cells (S5 Fig).

These data support the idea that a weaker OTI cell response could not be explained merely by death or lack of antigen presentation function of the *T*. *cruzi*-exposed BMDC-SIINFEKL and also suggest that the parasite-exposed BMDC rather actively trigger the *in trans* suppression of OTI CD8^+^ T cells.

### *T*. *cruzi*-exposed DC induce a proapoptotic phenotype in CD8^+^ T cells

**Fig 3:**
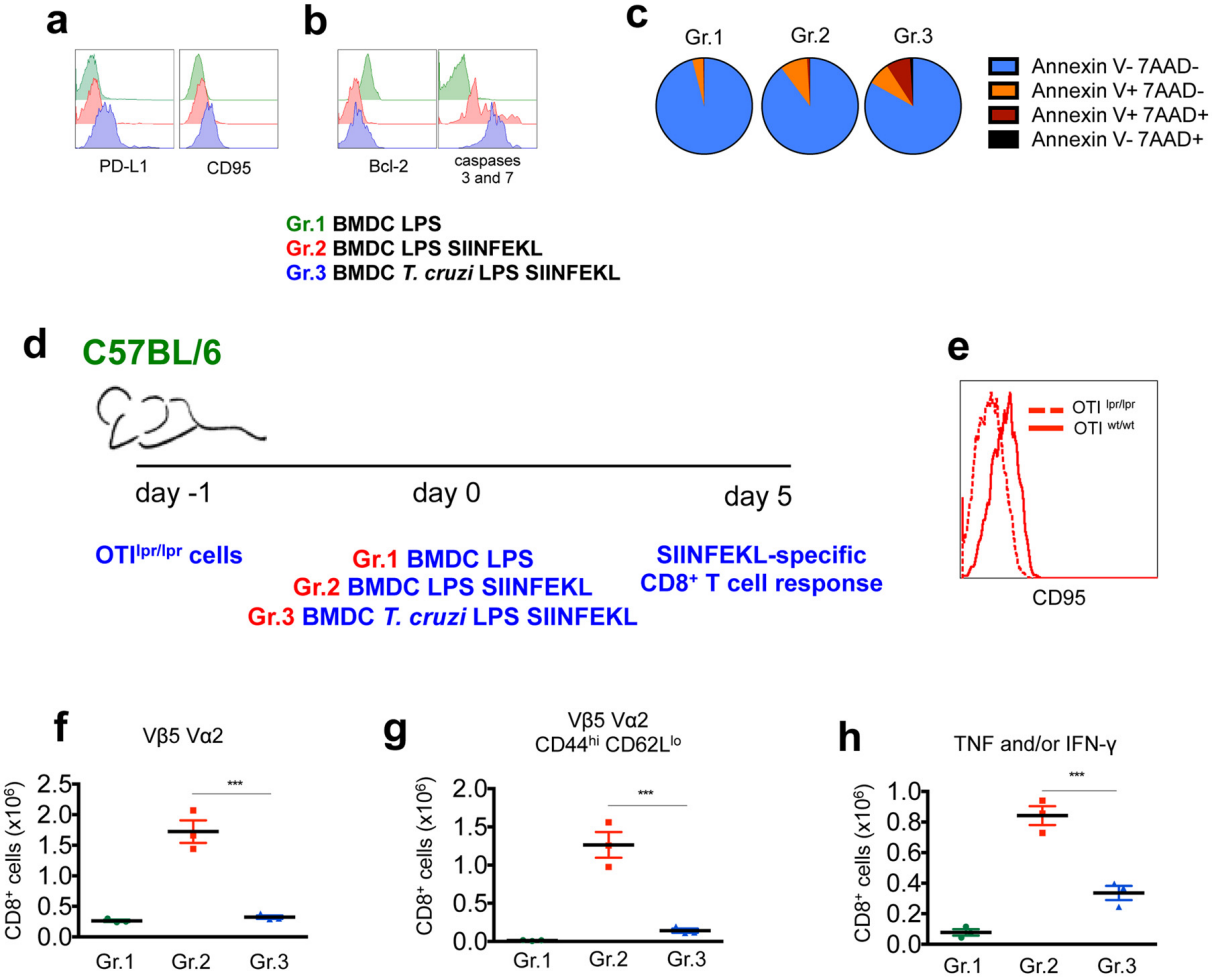
Proapoptotic phenotype of OTI CD8^+^ T cells upon stimulation with *T*. *cruzi*-exposed BMDC-SIINFEKL. 1 x 10^4^ OTI cells were adoptively transferred into C57BL/6 mice before the transfer of 5 x 10^5^ BMDC (Gr.1), 5 x 10^5^ BMDC-SIINFEKL (Gr.2) or 5 x 10^5^ *T*. *cruzi*-exposed BMDC-SIINFEKL (Gr.3). a- After 5 days, splenic CD8^+^ T cells were stained with H-2K^b^ SIINFEKL tetramers and mAbs to surface markers. b and c- To analyze viability of the tetramer-positive CD8^+^ T cells, fluorescent probes were used to stain Bcl-2 and the active forms of caspases 3 and 7 in parallel with staining for annexin V and 7AAD. d and e- 1 x 10^4^ OTI^lpr/lpr^ cells functionally deficient in CD95 were adoptively transferred into C57BL/6 mice before the transfer of 5 x 10^5^ BMDC (Gr.1), 5 x 10^5^ BMDC-SIINFEKL (Gr.2) or 5 x 10^5^ *T*. *cruzi*-exposed BMDC-SIINFEKL (Gr.3). f- Numbers of SIINFEKL-specific CD8^+^ T cells were determined by TCR Vα2 Vβ5 staining. g- The ability of naïve OTI cells to differentiate into effector cells was evaluated by CD44 and CD62L staining of TCR Vα2^+^ Vβ5^+^ CD8^+^ T cells. h- Spleen cells were restimulated *ex vivo* with SIINFEKL peptide and the numbers of TNF and/or IFN-γ-producing CD8^+^ T cells were assessed by ICS. Results are one of three separate experiments expressed as individual values and the mean ± SEM of each group. Asterisks indicate significant differences between groups (***P<0.001 One-way ANOVA followed by Tukey post-hoc test).

A detailed phenotypic characterization of the OTI CD8^+^ T cells from mice transferred with BMDC-SIINFEKL or *T*. *cruzi*-exposed BMDC-SIINFEKL was performed to further elucidate the mechanisms underlying suboptimal CD8^+^ T cell response. OTI cells collected from mice of each group were stained with CD8 antibody and H-2K^b^ SIINFEKL tetramers along with mAbs to surface markers related to activation, exhaustion, and migration of T cells, as well as nuclear staining of transcription factors related to T cell polarization and markers of cell viability. The major differences observed in tetramer-positive OTI CD8^+^ T cells from mice injected with *T*. *cruzi*-BMDC-SIINFEKL in comparison to BMDC-SIINFEKL were: higher expression of surface CD95 and PDL-1 (Fig 3a), lower staining of intracellular Bcl-2 and higher intracellular staining of active caspases 3 and 7 (Fig 3b), and higher frequency of Annexin V^+^ 7AAD^+^ cells (Fig 3c). For the other 36 markers analyzed the expression was, at most, slightly changed (S6 Fig).

The proapoptotic profile of OTI CD8^+^ T cells from animals that received *T*. *cruzi*-exposed BMDC-SIINFEKL resembles the augmented expression of CD95 by infection-induced CD8^+^ T cells specific for the immunodominant H-2K^b^-restricted epitope VNHRFTLV from *T*. *cruzi* [16]. Therefore, we further evaluated the role of CD95 in the suboptimal priming of CD8^+^ T cells. To this end, we generated OTI^lpr/lpr^ CD8^+^ T cells, which express mutated alleles that render CD95 non-functional. These OTI^lpr/lpr^ CD8^+^ T cells were employed in our *in vivo* model, as depicted in Fig 3d and 3e. Mice were transferred with OTI^lpr/lpr^ cells prior to immunization with BMDC-SIINFEKL or *T*. *cruzi-*exposed BMDC-SIINFEKL. The immune response of OTI^lpr/lpr^ CD8^+^ T cells from mice injected with *T*. *cruzi-*exposed BMDC-SIINFEKL was significantly lower than the BMDC-SIINFEKL counterpart, as measured by their numbers (Fig 3f), phenotype (CD44^hi^ CD62L^low^) (Fig 3g) and cytokine response (Fig 3h). These data thus indicate that CD95 expression by OTI cells is not the only factor responsible for the impaired response observed in mice transferred with *T*. *cruzi*-exposed BMDC-SIINFEKL.

### CD4^+^ T cells are required for the suboptimal response of CD8^+^ T cells upon *in vivo* stimulation with *T*. *cruzi*-exposed DC

**Fig 4:**
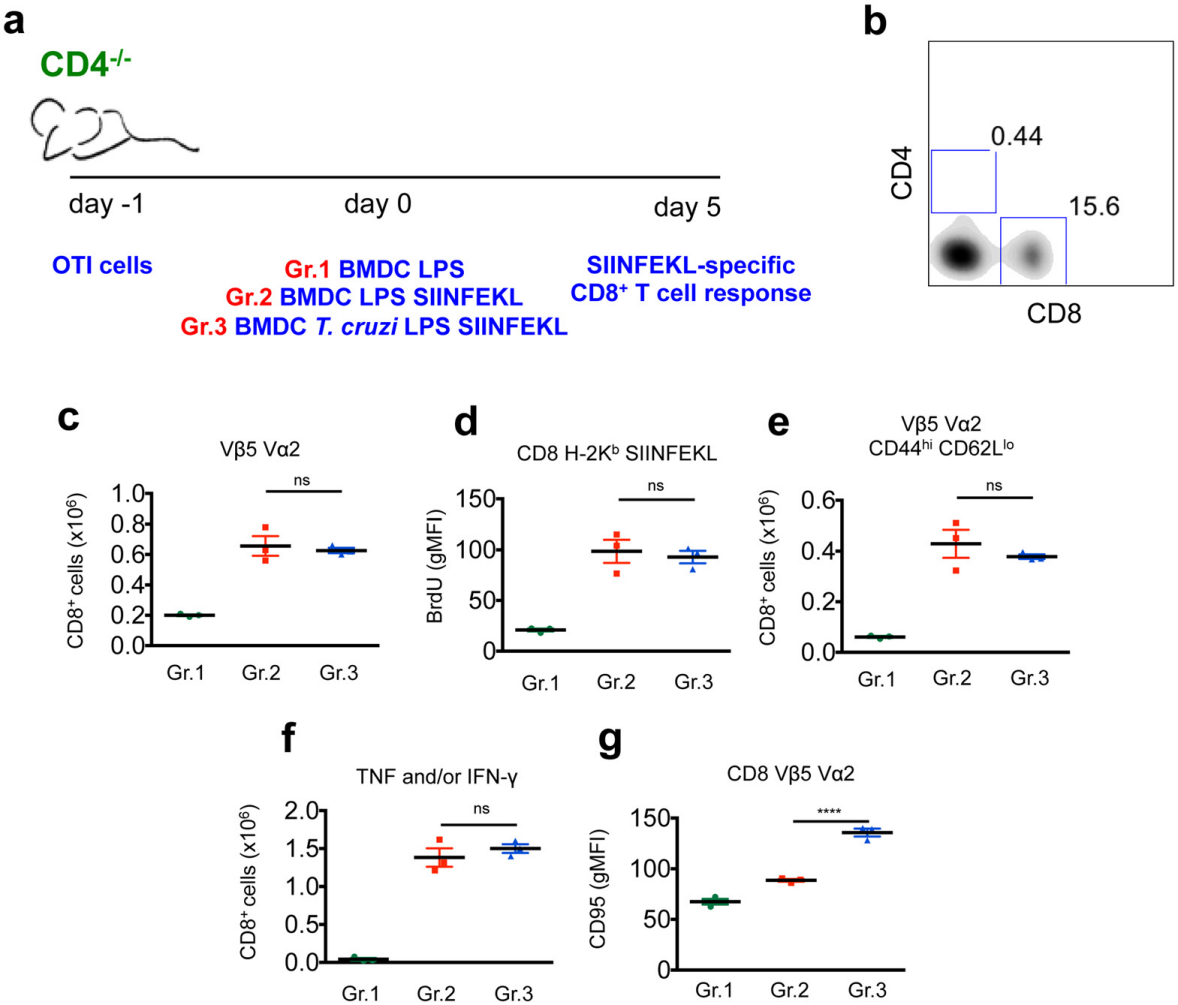
Optimal response of OTI CD8^+^ T cells upon *in vivo* stimulation of *cd4* ^-/-^ mice with *T*. *cruzi*-exposed BMDC-SIINFEKL. a and b- 1 x 10^5^ OTI cells were adoptively transferred into *cd4* ^-/-^ mice prior to transfer of 5 x 10^5^ BMDC (Gr.1), 5 x 10^5^ BMDC-SIINFEKL (Gr.2) or 5 x 10^5^ *T*. *cruzi*-exposed BMDC-SIINFEKL (Gr.3). The SIINFEKL-specific immune response was assessed after 5 days. c and d- The expansion of OTI cells was measured by numbers of TCR Vα2^+^ Vβ5^+^ CD8^+^ T cells or BrdU incorporation in H-2K^b^-SIINFEKL tetramer-stained cells. e- The ability of naïve OTI cells to differentiate into effector cells was evaluated by CD44 and CD62L staining of TCR Vα2^+^ Vβ5^+^ CD8+ T cells. f- Spleen cells were also restimulated *ex vivo* with SIINFEKL peptide and the numbers of TNF and/or IFN-γ-producing CD8^+^ T cells was assessed by ICS. g- CD95 staining on CD8^+^ Vα2^+^ Vβ5^+^ cells from each group reported as geometric mean of fluorescence intensity (gMFI). Results are one of three separate experiments expressed as individual values and the mean ± SEM of each group. Asterisks indicate significant differences between the indicated groups (****P<0.0001 One-way ANOVA followed by Tukey post-hoc test).

To determine whether CD4^+^ T cells mediate the suppression of the CD8^+^ T cell immune response in our model, we adoptively transferred OTI cells into *cd4* ^*-/-*^ animals and subsequently injected them with BMDC-SIINFEKL or *T*. *cruzi*-exposed BMDC-SIINFEKL (Fig 4a and 4b). In the absence of CD4^+^ T cells, the proliferation (Fig 4c and 4d), phenotype (CD44^hi^ CD62L^low^) (Fig 4e) and cytokine response (Fig 4f) of OTI cells from animals that received BMDC-SIINFEKL (Gr. 2) or *T*. *cruzi*-exposed BMDC-SIINFEKL (Gr. 3) were comparable. Of note, Vα2^+^Vβ5^+^ CD8^+^ T cells from mice immunized with *T*. *cruzi*-exposed BMDC-SIINFEKL showed upregulated CD95 expression (Fig 4g), which further supports the notion that the impaired response of OTI CD8^+^ T cells is not (at least exclusively) dependent on CD95 expression.

To confirm these findings, we also transferred purified splenic CD4^+^ T cells isolated from mice injected with *T*. *cruzi*-exposed BMDC into mice carrying OTI cells and further injected them with BMDC-SIINFEKL (S7a Fig). After 5 days, we assessed the proliferation (S7b and S7c Fig), phenotype (CD44^hi^ CD62L^low^) (S7d Fig) and cytokine response (S7e Fig) of OTI cells in these mice. We found that suppression of OTI CD8^+^ T cell priming could be transferred to non-infected mice by the specific injection of *T*. *cruzi-*induced CD4^+^ T cells.

### CD4^+^ Foxp3^+^ T cells induced by *T*. *cruzi*-exposed DC suppress the priming of CD8^+^ T cells *in vivo*

**Fig 5:**
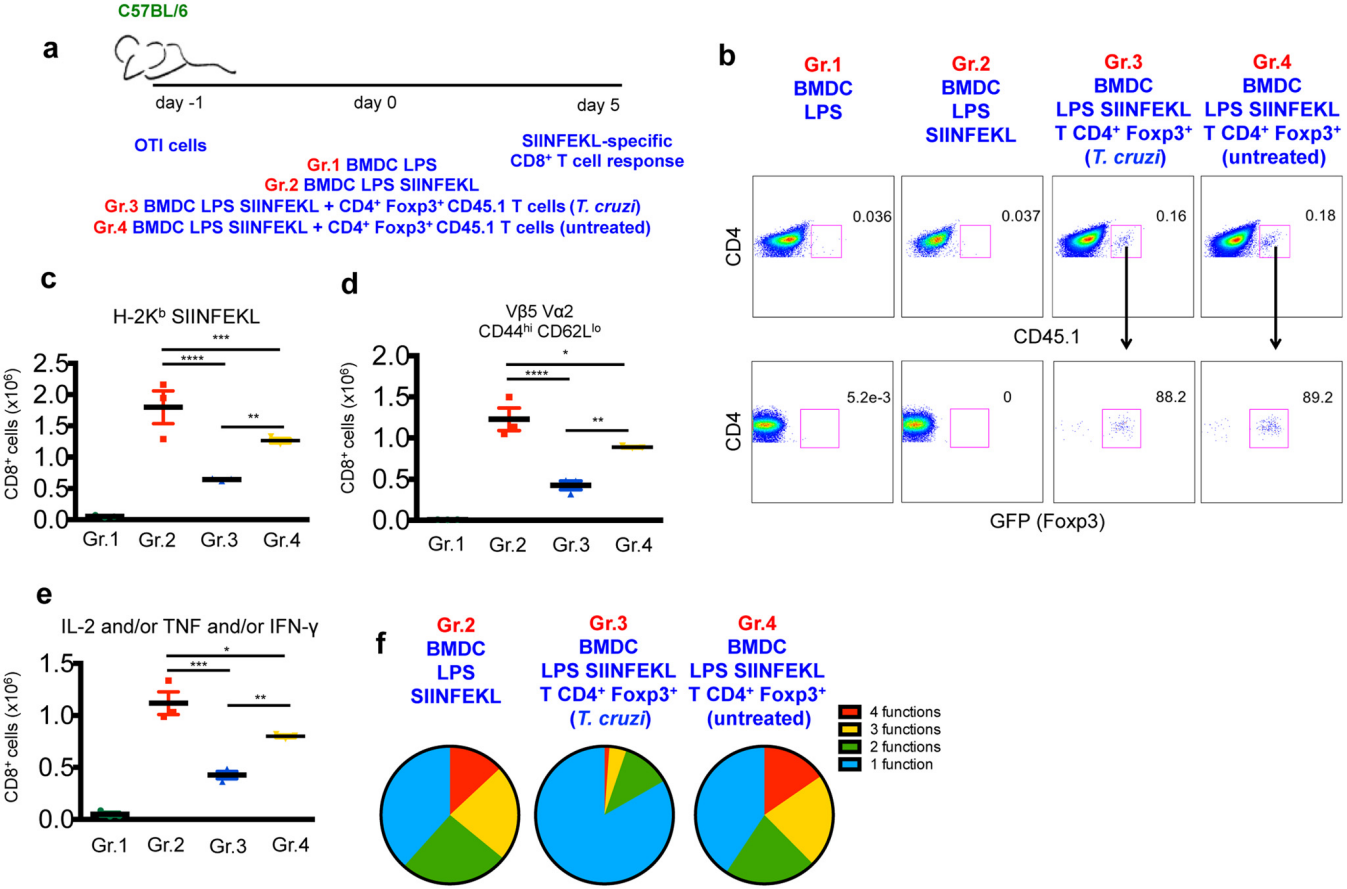
CD4^+^Foxp3^+^ cells induced by *T*. *cruzi*-exposed BMDC mediate the suppression of OTI CD8^+^ T cell priming. a and b- 5 x 10^5^ untreated BMDC or 5 x 10^5^ *T*. *cruzi*-exposed BMDC were adoptively transferred into Foxp3-GFP CD45.1^+^ mice and the splenic CD4^+^GFP^+^ cells were sorted after 5 days. These cells (1 x 10^6^) were transfer into C57BL/6 mice on the same day of OTI cell transfer (1 x 10^4^ cells) and 24 h before 5 x 10^5^ BMDC-SIINFEKL transfer. The SIINFEKL-specific immune response was assessed after 5 days. c- The numbers of SIINFEKL-specific CD8^+^ T cells were determined by H-2K^b^-SIINFEKL tetramer staining. d- The ability of naïve OTI cells to differentiate into effector cells was evaluated by CD44 and CD62L staining of TCR Vα2^+^ Vβ5^+^ CD8^+^ T cells. e- Spleen cells were restimulated *ex vivo* with SIINFEKL peptide and the numbers of IL-2 and/or TNF and/or IFN-γ-producing CD8^+^ T cells were determined by ICS. f- Proportion of polyfunctional CD8^+^ T cells stained for CD107a and/or one, two or three cytokines (IL-2, IFN-γ and TNF) combined after restimulation with SIINFEKL peptide. Results are one of two separate experiments expressed as individual values and the mean ± SEM of each group. Asterisks indicate significant differences between the indicated groups (*P<0.05, **P<0.01, ***P<0.001, ****P<0.0001 One-way ANOVA followed by Tukey post-hoc test).

To further investigate the involvement of CD4^+^ T cells in the suppression of CD8^+^ T cell priming, we transferred either untreated BMDC or *T*. *cruzi*-exposed BMDC into congenic mice expressing GFP under control of the Foxp3 promoter (Foxp3-GFP CD45.1 mice). After 5 days, we sorted the splenic CD4^+^ Foxp3^+^ CD45.1^+^ congenic cells and transferred them (1 x 10^6^ cells/mouse) into C57BL/6 (CD45.2^+^) mice that had received OTI CD8^+^ T cells on the day before. These animals were then injected with BMDC previously stimulated with LPS and loaded or not with SIINFEKL peptide, and the response of cognate OTI CD8^+^ T cells was assessed after 5 days, as outlined in Fig 5a. As indicated in Fig 5b, the frequencies of CD45.1^+^ and GFP^+^ cells found in the spleen of the recipient C57BL/6 mice 5 days after the adoptive transfer were similar between the group that received CD4^+^ Foxp3^+^ cells induced in the presence (Gr.3) or absence (Gr.4) of *T*. *cruzi*. Although both groups received equal numbers of Foxp3^+^ cells that exerted regulatory functions over OTI cell priming, the expansion (Fig 5c), activation (CD44^hi^ CD62L^low^) (Fig 5d) and cytokine response (Fig 5e) of OTI CD8^+^ T cells was significantly more impaired upon transfer of CD4^+^ Foxp3^+^ cells induced in the presence of *T*. *cruzi*-exposed BMDC as compared to the transfer of CD4^+^ Foxp3^+^ cells isolated from mice previously injected with BMDC unexposed to the parasite. Additionally, when the function of the responding OTI CD8^+^ T cells after stimulation with SIINFEKL peptide was assessed by the expression of CD107a, IL-2, TNF, or IFN-γ combined, we observed that although CD4^+^ Foxp3^+^ T cells induced in the absence of *T*. *cruzi* suppressed the magnitude of the cytokine response of CD8^+^ T cells, the remainder responding cells were still polyfunctional, whereas CD8^+^ T cells suppressed by CD4^+^ Foxp3^+^ T cells induced by *T*. cruzi-exposed BMDC were mostly positive for only one out of the four markers analyzed (Fig 5f).

**Fig 6:**
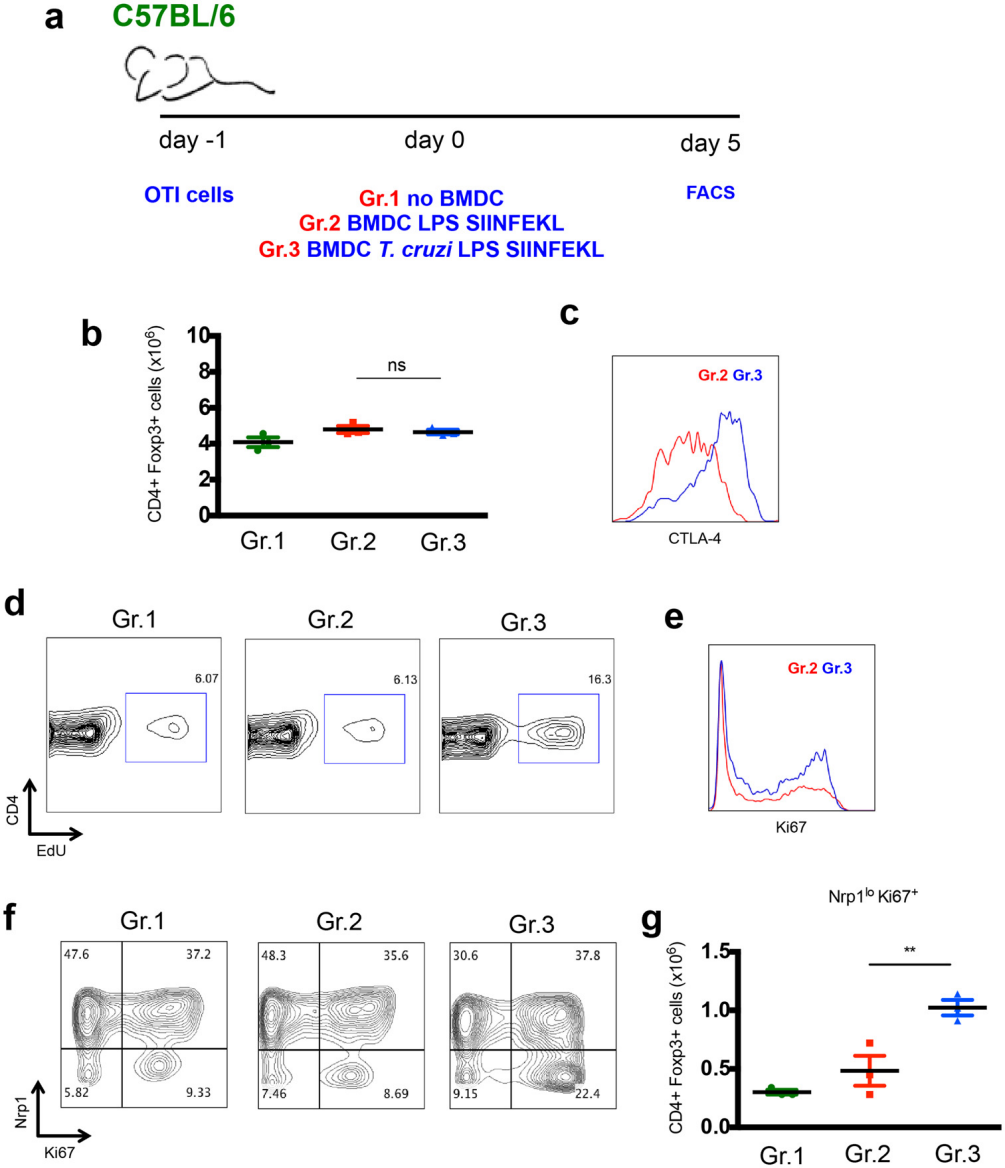
Induction of Treg expressing high levels of CTLA-4 after adoptive transfer of *T*. *cruzi*-exposed BMDC. a- 1 x 10^4^ OTI cells were adoptively transferred into C57BL/6 mice prior to transfer of 5 x 10^5^ BMDC exposed to LPS and loaded with SIINFEKL peptide (Gr. 2) or 5 x 10^5^ BMDC previously exposed to *T*. *cruzi* and LPS and loaded with SIINFEKL peptide (Gr. 3). Control animals did not receive BMDC transfer (Gr.1). b- After 5 days, the number of splenic CD4^+^Foxp3^+^ cells was determined. c- CTLA-4 staining gated in CD4^+^Foxp3^+^ cells from Gr.2 and Gr.3. d- Mice were injected with EdU 4 h before FACS and its incorporation in DNA of CD4^+^Foxp3^+^ cells was assessed. e- proliferation of CD4^+^Foxp3^+^ cells was also assessed by Ki67 staining. f- Ki67 and Nrp-1 staining gated in CD4^+^Foxp3^+^ cells. g- Total numbers of splenic CD4^+^ Foxp3^+^ Nrp-1^lo^ Ki67^+^ cells. Results expressed as individual values and the mean ± SEM of each group. Asterisks indicate significant differences between groups (**P<0.01, One-way ANOVA followed by Tukey post-hoc test).

We further characterized the CD4^+^ Foxp3^+^ T cells present in mice adoptively transferred with BMDC exposed or not to *T*. *cruzi*, as depicted in Fig 6a. Consistent with the data presented in Fig 5, *T*. *cruzi*-exposed BMDC did not induce any augment in total numbers of splenic CD4^+^ Foxp3^+^ cells (Fig 6b). Nonetheless, upon transfer of *T*. *cruzi*-exposed BMDC, the CD4^+^ Foxp3^+^ cells upregulated CTLA-4 (Fig 6c) and were induced to proliferate, as indicated by EdU incorporation (Fig 6d) and Ki67 staining (Fig 6e). We also characterized these cells by neuropilin-1 (Nrp-1) staining, a marker highly expressed in thymic-derived Treg, but downregulated in Treg induced in the periphery [19,20]. As shown in Fig 6f and 6g, the frequency and numbers of Nrp-1^lo^ Ki67^+^ Treg increased in animals receiving *T*. *cruzi*-exposed BMDC. Altogether, these data suggest that *T*. *cruzi* is able to induce Treg in the periphery, and the enhanced suppressive function of Treg correlated with high levels of CTLA-4 expression.

### Involvement of CTLA-4 and TGF-β, but not IL-10, in the suppression of CD8^+^ T cell priming

We further investigated possible mechanisms of CD8^+^ T cell regulation in our model. IL-10, which was highly expressed by *T*. *cruzi*-exposed BMDC (Fig 2), is an immune regulatory cytokine described as an inhibitor of CD8^+^ T cell immune responses [21]. We evaluated the *in vivo* activation of OTI cells using *il10* ^*-/-*^ mice as recipients and BMDC donors (S8a and S8b Fig). Similar to the observed in C57BL/6 WT mice, the response of OTI CD8^+^ T cells in *il10* ^*-/-*^ mice injected with *T*. *cruzi-*exposed BMDC-SIINFEKL was significantly lower when compared to the OTI cell response of mice injected with BMDC-SIINFEKL, as measured by TCR and H-2K^b^ SIINFEKL tetramer staining (S8c and S8d Fig), effector phenotype (CD44^hi^ CD62L^low^) (S8e Fig) and cytokine response (S8f Fig).

**Fig 7:**
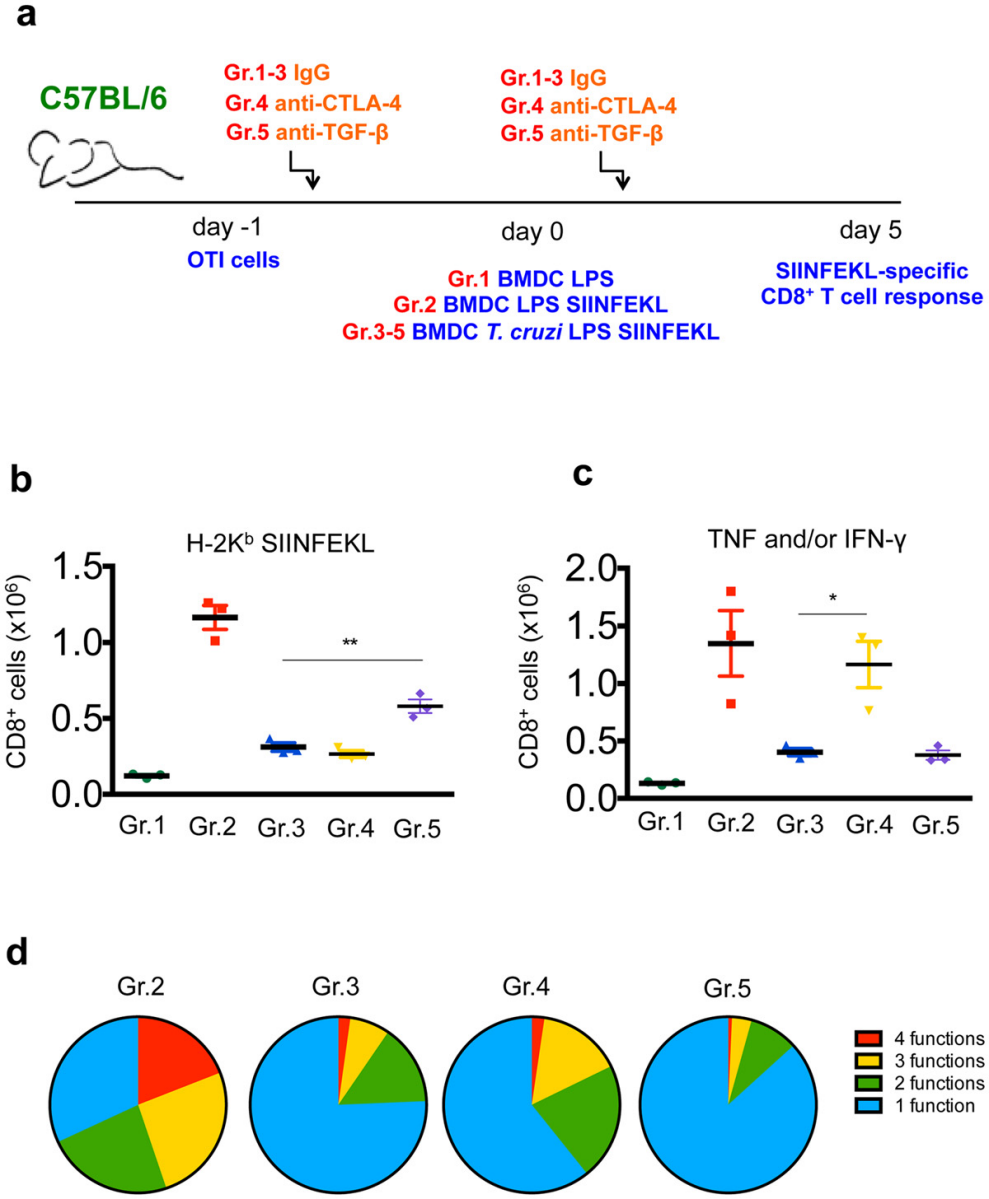
CTLA-4 and TGF-β contribute to the suppression of OTI CD8^+^ T cell priming induced by *T*. *cruzi*-exposed BMDC. a- 1 x 10^4^ OTI cells were adoptively transferred into C57BL/6 mice treated with IgG or blocking antibodies against CTLA-4 or TGF-β. These mice were transferred with 5 x 10^5^ BMDC (Gr.1), 5 x 10^5^ BMDC-SIINFEKL (Gr.2) or 5 x 10^5^ *T*. *cruzi*-exposed BMDC-SIINFEKL (Gr.3). The SIINFEKL-specific immune response was assessed after 5 days. b- The numbers of SIINFEKL-specific CD8^+^ T cells were determined by H-2K^b^-SIINFEKL tetramer staining. c- Spleen cells were also restimulated *ex vivo* with SIINFEKL peptide and the numbers of TNF and/or IFN-γ-producing CD8^+^ T cells were assessed by ICS. d- The proportion of polyfunctional cells simultaneously stained for CD107a, IL-2, TNF and IFN-γ was determined by Boolean analysis. Results are one of two separate experiments expressed as individual values and the mean ± SEM of each group. Asterisks indicate significant differences between the indicated groups (*P<0.05, **P<0.01 One-way ANOVA followed by Tukey post-hoc test).

We also addressed the involvement of CTLA-4 (as suggested by the phenotype reported in Fig 6) and TGF-β in the suppression of OTI CD8^+^ T cell priming by *T*. cruzi-exposed BMDC loaded with SIINFEKL peptide. To this end, we treated the recipient mice with CTLA-4 and TGF-β blocking antibodies (clones 9D9 and 1D11.16.8, respectively) as outlined in Fig 7a. We observed that the treatment with clone 1D11.16.8 to block TGF-β partially restored the ability of OTI CD8^+^ T cells to proliferate upon *in vivo* stimulation with *T*. *cruzi*-exposed BMDC-SIINFEKL, as measured by tetramer staining (Fig 7b), whereas the treatment with 9D9 CTLA-4-blocking antibody rescued the ability of OTI CD8^+^ T cells to produce TNF and IFN-γ (Fig 7c). Accordingly, 9D9 treatment partially recovered the polyfunctionality of OTI CD8^+^ T cells, as shown by the frequencies of cells simultaneously stained for TNF, IFN-γ, IL-2 and/or CD107a (Fig 7d).

Therefore, we concluded that the suppression of OTI CD8^+^ T cell priming by DC exposed to *T*. *cruzi* is independent of IL-10, but may be partially mediated by CTLA-4 and TGF-β.

### Depletion of Foxp3^+^ cells leads to optimal priming of specific CD8^+^ T lymphocytes during *T*. *cruzi* infection

**Fig 8:**
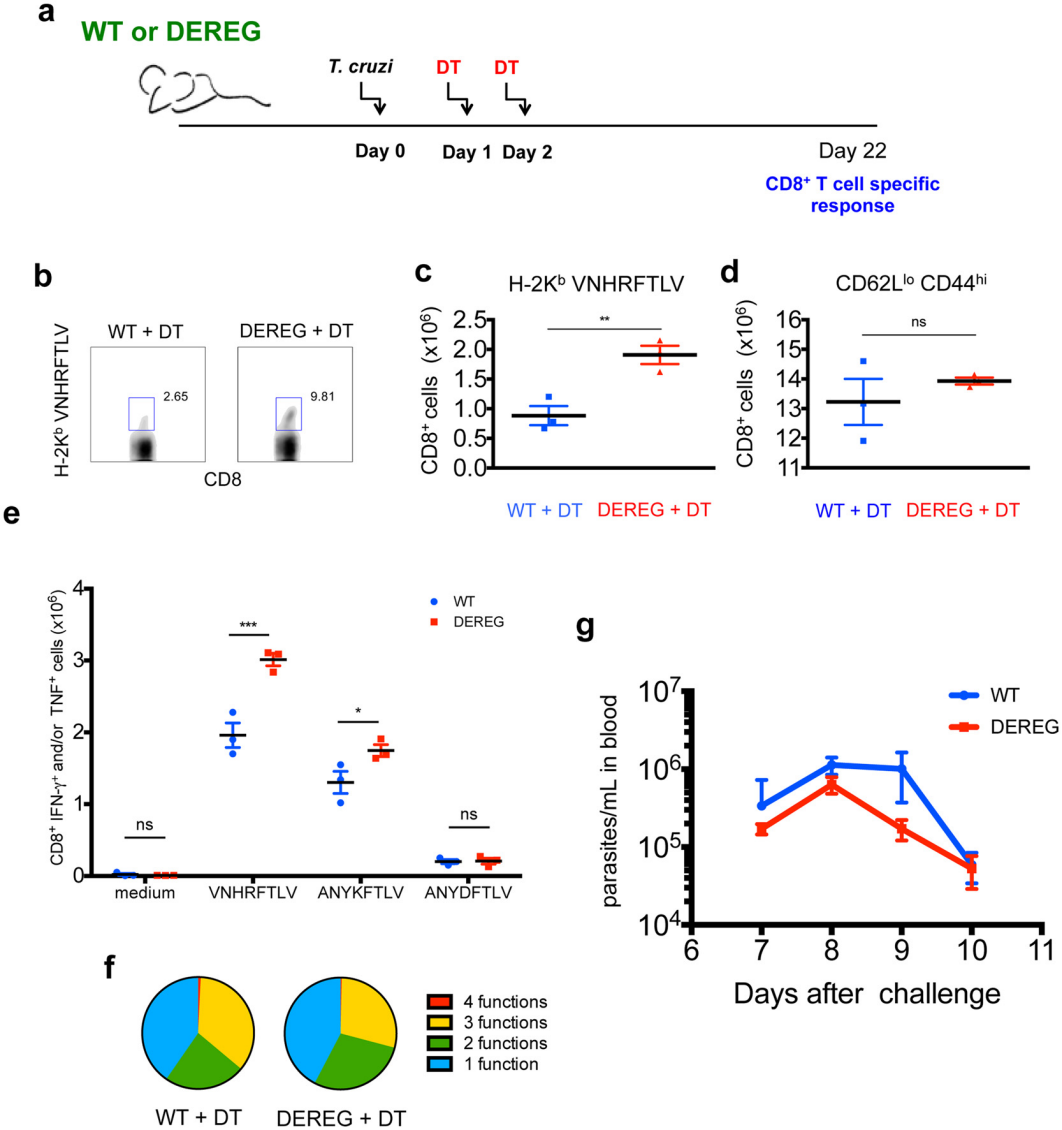
Depletion of Foxp3^+^ cells improves the priming of specific CD8^+^ T cells upon *T*. *cruzi* infection. a- DEREG mice or their WT littermates were infected with *T*. *cruzi* and i.p. treated with 0.5 μg diphtheria toxin on the two following days. On day 22 after infection, the response of specific CD8^+^ T cells was assessed. b and c- The numbers of SIINFEKL-specific CD8^+^ T cells were determined by H-2K^b^-SIINFEKL tetramer staining. d- Numbers of CD8^+^ CD44^hi^ CD62L^low^ cells. e- Spleen cells were restimulated *ex vivo* with VNHRFTLV, ANYKFTLV and ANYDFTLV peptides corresponding to H-2K^b^-restricted *T*. *cruzi* epitopes and the numbers of TNF and/or IFN-γ-producing CD8^+^ T cells was assessed by ICS. f- The proportion of polyfunctional cells simultaneously stained for CD107a, IL-2, TNF and IFN-γ was determined by Boolean analysis. g- parasitemia in blood was assessed and values were log-transformed. Values from peak parasitemia (day 9) were significantly different between DEREG and WT mice P<0.05. Results are expressed as individual values and the mean ± SEM of each group. Asterisks indicate significant differences between the indicated groups (*P<0.05, **P<0.01, ***P<0.001 One-way ANOVA followed by Tukey post-hoc test).

To extend the findings described above to the context of *T*. *cruzi* infection, we challenged DEREG mice (which express the diphtheria toxin receptor under control of Foxp3 promoter) with the parasite. As control group, we infected their WT littermates with *T*. *cruzi*. All animals were i.p. treated with diphtheria toxin (DT) on the two consecutive days after infection, as outlined in Fig 8a. After 22 days, the response of CD8^+^ T cells specific to *T*. *cruzi* epitopes was assessed. The numbers of CD8^+^ T cells specific to the immunodominant H-2K^b^-restricted epitope VNHRFTLV from *T*. *cruzi* were significantly increased in the group depleted of Foxp3^+^ cells early after infection, as measured by H-2K^b^-VNHRFTLV pentamer staining (Fig 8b and 8c), although the numbers of total CD8^+^ CD44^hi^ CD62L^low^ cells was not significantly altered by early Foxp3^+^ cell depletion (Fig 8d). Accordingly, the cytokine response of CD8^+^ T cells specific to the epitopes VNHRFTLV and ANYKFTLV from *T*. *cruzi* was higher in the group subjected to depletion of Foxp3^+^ cells, as measured by ICS, whereas the response to the subdominant H-2K^b^-restricted epitope ANYDFTLV was equally low in both groups (Fig 8e). However, the polyfunctionality of CD8^+^ T cells *ex vivo*-stimulated with VNHRFTLV peptide on day 22 post-infection was not enhanced in DT-treated DEREG mice (Fig 8f), even though these mice were able to control *T*. *cruzi* infection better than their WT littermates, as measured by parasitemia in blood (Fig 8g). Jointly, these findings thus suggest a role of Foxp3^+^ cells in the suboptimal priming of specific CD8^+^ T cells early after *T*. *cruzi* infection.

## Discussion

Dendritic cells initiate CD8^+^ T cell-mediated immune responses in different experimental models of infection with viruses, bacteria and protozoans [22–27]. Here, we aimed at clarifying whether exposure to *T*. *cruzi* would interfere with antigen presentation function of DC. We observed that *T*. *cruzi* exposure drastically impaired the ability of BMDC-SIINFEKL to prime OTI cells *in vivo*, but not *in vitro*. Our results greatly diverged from a recent study using a similar approach for OTI CD8^+^ T cell *in vivo* priming in the context of LCMV or *Listeria monocytogenes* infection, where presence of these pathogens did not inhibit and even improved the avidity of cognate OTI cells [28]. These discrepant observations are compatible with the fact that acute infections with these microorganisms elicit a rapid and efficient CD8^+^ T cell immune response that in most cases cures the host. In contrast, *T*. *cruzi* infection elicits a delayed and suboptimal immune response that is unable to protect susceptible mice from death and allows the establishment of a chronic infection in resistant mouse strains [15,16].

We observed that *T*. *cruzi*-exposed BMDC-SIINFEKL-induced suboptimal priming of OTI cells is unlikely to be mediated by its direct effects on CD8^+^ T cells, as it did not take place in *cd4* ^-/-^ mice. The observation that the priming of OTI CD8^+^ T cells deficient in CD95 was still impaired in the presence of *T*. *cruzi*-exposed BMDC-SIINFEKL also favors this assumption.

Furthermore, the experiments using *cd4* ^-/-^ mice that allowed the observation of unaltered OTI cell priming by *T*. *cruzi*-exposed BMDC in comparison to parasite-unexposed BMDC are in agreement with our *in vitro* observations that ruled out several other possibilities impacting DC antigen presentation function, such as lack of MHCI-peptide complexes (signal I), co-stimulatory molecules (signal 2), or inflammatory cytokines (signal 3).

On the other hand, adoptive transfer of CD4^+^ Foxp3^+^ T cells from mice stimulated with *T*. *cruzi*-exposed BMDC was able to reproduce the suppression of OTI CD8^+^ T cell priming *in vivo*, in absence of infection.

It is reasonable to speculate that host cells infected with *T*. *cruzi* may trigger Treg-mediated suppression of CD8^+^ T cell priming. Because in our experimental model for *in vivo* priming of OTI cells we transferred *T*. *cruzi* intracellular parasites within BMDC, and these BMDC most likely have not released parasites during the short term of our experimental set up (and we were never able to find parasites in blood of mice from Gr.3), presumably the transferred BMDC were the major cell subset responsible for the induction of Treg, thus ensuing CD8^+^ T cell priming suppression. Nonetheless, we did not completely ruled out the possibility that host cells other than the transferred BMDC might also have contributed to this induction.

Treg isolated from mice that received *T*.*cruzi*-exposed BMDC were significantly more suppressive than equal numbers of CD4^+^ Foxp3^+^ cells isolated from *T*. *cruzi*-unexposed animals, which correlated with higher levels of CTLA-4 expression. Moreover, proliferation and downregulation of Nrp-1 indicated that in mice injected with *T*. *cruzi*-exposed BMDC there was induction of Treg in the periphery. The precise mechanism of Treg induction in our model remains to be elucidated. In this regard, similarly to our observations, Poncini and colleagues recently reported on the induction of CD4^+^ Foxp3^+^ cells by DC upon *T*. *cruzi* infection in a galectin-1-dependent fashion [29]. The group suggested that this lectin confers tolerogenic properties to dendritic cells, and mice deficient in galectin-1 presented increased response of CD8^+^ T cells following *T*. *cruzi* infection [29].

Another open question regarding the induction of Treg by *T*. *cruzi* refers to their TCR specificity and affinity. Although we were unable to determine the specificity and affinity of the Treg TCR repertoire in mice injected with *T*. *cruzi*-exposed BMDC, their phenotype could suggest that induction was antigen-dependent. Favoring this idea, injection of recombinant *T*. *cruzi* amastigote antigen SSP4 was reported to induce Treg with enhanced suppressive function in BALB/c mice [30]. Although the induction of Treg by *T*. *cruzi* may be antigen-specific, the *in trans* suppression of OTI CD8^+^ T cell priming observed when *T*.*cruzi*-exposed BMDC and SIINFEKL-loaded BMDC were co-injected clearly demonstrated that Treg suppressive functions reported here are not antigen-specific. This is in line with other classic reports and recent findings suggesting that, in comparison to effector T cells, Treg are largely unresponsive to TCR stimulation, but highly sensitive to cytokines mediating intercellular communication [31].

Most mechanisms of T cell regulation were described in CD4^+^ T cell-mediated responses, whereas their extension to CD8^+^ T cells is less clear. Recent studies have highlighted that TGF-β mediates the immunosuppression of CD8^+^ T cells by elevating miR-23a and downregulating Blimp-1, or by upregulating Foxp1 [32–34]. When CD4-DNRII mice (lacking TGF-βRII kinase domain in both CD4^+^ and CD8^+^ T cells) were infected with *T*. *cruzi*, parasite-specific CD8^+^ T cells proliferated more, but remained functionally impaired [9]. Here, using the model of OTI CD8^+^ T cell priming by *T*. *cruzi*-exposed BMDC-SIINFEKL during treatment with antibodies to block TGF-β, we also observed augmented proliferation of OTI CD8^+^ T cells, although their capacity to produce effector cytokines persisted compromised.

In addition, CTLA-4 has been suggested as an important mediator of CD8^+^ T cell regulation, both in mice and humans [35,36]. For instance, it has been proposed that memory CD8^+^ T cell quiescence relies on its active suppression by Treg in a CTLA-4-dependent way [35]. Here, we showed that *T*. cruzi-induced Treg significantly upregulated CTLA-4, and antibodies blocking the inhibitory activity of CTLA-4 partially restored the magnitude and polyfunctionality of OTI CD8^+^ T cell cytokine response after adoptive transfer of *T*. *cruzi*-exposed BMDC-SIINFEKL. In line with this, administration of anti-CTLA-4 antibodies during infection with *T*. *cruzi* has been shown to improve CD8^+^ T cell-mediated immunity [37,38]. Ideally, however, the cell-intrinsic role of CTLA-4 specifically expressed by the subset of Treg induced by *T*. *cruzi* would confirm the actual contribution of this molecule to the suppression of CD8^+^ T cell priming.

Collectively, our data indicate that CD4^+^ Foxp3^+^ Treg induced by *T*. *cruzi* are able to suppress the induction of CD8^+^ T cell responses. These findings are not in accordance with previous studies using antibodies to deplete CD25^+^ cells, which suggested that Treg did not impact the onset of the CD8^+^ T cell-mediated immunity during *T*. *cruzi* infection [39,40]. In line with these previous reports, we also observed the suppression of CD8^+^ T cell priming upon transfer of *T*. *cruzi*-exposed BMDC-SIINFEKL when we depleted CD25^+^ cells with PC61 antibody (S9 Fig). Incomplete depletion of CD25^+^ Foxp3^+^ cells by antibodies, as well as the putative contribution of CD25^-^ Foxp3^+^ cells may explain these observations.

The increase in numbers and cytokine response of *T*. cruzi-specific CD8^+^ T cells upon Foxp3^+^ T cell depletion *in vivo* soon after infection and the enhanced protection of these mice extend our results from the model of OTI CD8^+^ T cell priming and sustains the role of Treg in the suppression of CD8^+^ T cells. These data are also in line with recent studies showing that Foxp3^+^ Treg effectively maintained CD8^+^ T cell exhaustion during chronic infection with LCMV and inhibited the activation of CD8^+^ T cells by DC [41].

In conclusion, the data presented here is consistent with a model in which *T*. *cruzi*- infected DC suppress rather than induce specific CD8^+^ T cell immunity. This immune evasion mechanism might be relevant to the establishment of a chronic phase of infection and relies on the induction of regulatory CD4^+^ Foxp3^+^ T cells that actively suppress CD8^+^ T cell priming and curtail their functionality.

## Methods

### Ethics statement

This study was carried out in strict accordance with the recommendations in the Guide for the Care and Use of Laboratory Animals of the Brazilian National Council of Animal Experimentation (http://www.cobea.org.br/). The protocol was approved by the Committee on the Ethics of Animal Experiments of the Institutional Animal Care and Use Committee at the Federal University of Sao Paulo (Id # CEP 0426/09).

### Mice and parasites

Female 8- to 12-week-old animals were used in all experiments. C57BL/6 mice were purchased from CEDEME (Federal University of São Paulo). C57BL/6-Tg(TcraTcrb)1100 Mjb/J mice with transgenic OTI CD8 T cells were purchased from Jackson Laboratories (Bar Harbour, ME) and bred at CEDEME. B6.MRL-*Fas* ^*lpr*^ */J* and B6.129P2-*Il10* ^*tm1Cgn*^/J mice originally purchased from The Jackson Laboratory were bred and provided by Dr. Gustavo Amarante Mendes (University of São Paulo). B6.MRL-*Fas* ^*lpr*^ */J* mice were crossed with C57BL/6-Tg(TcraTcrb)1100 Mjb/J at CEDEME to generate animals with transgenic OTI^lpr/lpr^ CD8 T cells. B6.129S2-Cd4^tm1Mak^/J mice were originally purchased from The Jackson Laboratory and provided by Dr. Alexandre Keller (Federal University of São Paulo). Mice with a bicistronic insertion of the reporter gene encoding eGFP in the Foxp3 locus (Foxp3-GFP) were generated in the laboratory of Vijay Kuchroo (Harvard Medical School) [42]. These animals were crossed with Cby.SJL(B6)-*Ptprc* ^a^/J (CD45.1) mice from the Jackson Laboratory for use in the experiments. Mice expressing the simian receptor of diphtheria toxin and eGFP under control of Foxp3 promoter (DEREG) were purchased from the Jackson Laboratory.

Animals were injected *i*.*v*. with 1×10^4^ transgenic OTI CD8 T cells one day before the *i*.*v*. transfer of 5×10^5^ BMDC. For *in vivo* cellular proliferation assays, mice were injected *i*.*p*. at the same day of BMDC transfer with 200μL 10 mg/mL BrdU diluted in PBS. BrdU treatment was repeated every 48 h until the end of the experiment. EdU (200μL 5 mg/mL) was injected i.v. 4 h before FACS. For CD25^+^ cell depletion, mice were injected *i*.*p*. with 0.5 mL PC61 ascite fluid every 48 h during the experiment, with the first treatment 2 days prior to BMDC transfer. To inhibit CTLA-4 function *in vivo*, mice were treated i.p. every 48h with 1mg of 9D9 antibody (BioXCell). To neutralize TGF-β function *in vivo* mice were treated with 2mg of 1D11.16 antibody (BioXCell) every 72h. Control groups were similarly treated with rat polyclonal IgG (Sigma). Bloodstream trypomastigotes of the Y strain of *T*. *cruzi* were cultured *in vitro* in LLC-MK2 cells (ATCC) for *in vitro* incubation with BMDC, or obtained from mice infected 7 days earlier and injected s.c. in the base of the tail (1×10^4^ parasites/animal) for *in vivo* challenge. The mice were i.p. treated with 0.5 μg diphtheria toxin diluted in 200 μL PBS on the two consecutive days following challenge.

### BMDC generation

Bone marrow cells were flushed from femurs and cultured *in vitro* in RPMI 1640 supplemented with 10 mM Hepes, 0.2% sodium bicarbonate, 59 mg/L of penicillin, 133 mg/L of streptomycin, 10% Hyclone fetal bovine serum, 2 mM L-glutamine, 1 mM sodium pyruvate, 55μM 2-mercaptoethanol and 20 ng/mL GM-CSF (R&D Systems) at a concentration of 2×10^5^ cells/mL. After 4 days in culture, half of the volume was replaced by fresh medium. At day 6, the cells were exposed for additional 24 h to tissue culture trypomastigotes of *Trypanosoma cruzi* at the ratio of 3 parasites/cell or left unexposed. At day 7, BMDC were stimulated with 1 μg/mL LPS (Sigma) for 6 h, washed, incubated with 2μM SIINFEKL peptide for 1 h, washed, and transferred into mice. Control BMDC were stimulated with LPS only. For the RT-PCR and the *in vitro* antigen presentation Elispot assay BMDC were exposed to *T*. *cruzi* for 24 h or 48 h, respectively, or unexposed. Where stated, BMDC were stimulated with LPS 1 μg/mL for the last 6 h in culture.

### Cell viability assays

In order to assess the viability of BMDC and lymphocytes, cells were prepared and stained according to manufacturer’s instructions using PE Annexin V Apoptosis Detection Kit I (BD), Vybrant FAM Caspase-3 and -7 Assay Kit (Molecular Probes), and FITC Hamster anti-mouse Bcl-2 sets (BD). As a positive control for apoptosis induction, cells were incubated with actinomycin D 5 μg/mL for 5 h.

### Immunological assays

Synthetic peptides SIINFEKL, VNHRFTLV and ANYKFTLV were purchased from Genscript (Piscataway, New Jersey). The biotinylated tetramer H-2K^b^-SIINFEKL was purchased from ProImmune Inc. (Oxford, UK).

Tetramer staining was performed before other FACS staining per manufacturer’s instructions. The intracellular cytokine staining of spleen cells was performed after 6 h of *ex vivo* restimulation with SIINFEKL 10 μM as described earlier [16]. CTLA-4 staining was performed following ICS procedures. The intranuclear staining of transcription factors was performed following the manufacturer’s instructions using Foxp3 Fixation/Permeabilization Concentrate and Diluent and Permeabilization Buffer (eBioscience). BrdU staining was performed with BD FITC BrdU Flow Kit according to the manufacturer’s instructions. EdU staining was performed with Click it Plus EdU Alexa 488 kit (Molecular Probes). Samples were acquired immediately after staining in a BD FACSCanto II flow cytometer and analyzed in FlowJo 8.7 (Tree Star).

For flow cytometry stainings the following antibodies were used: CD11c APCCy7 (HL3, BD), CD40 APC (2/23, BD), CD80 PerCP (16-10A1, BD), CD86 PECy7 (GL1, BD), H-2K^b^ FITC (AF6-88.5, BD), I-A^b^ PE (AF6-120.1, BD), CD8 PerCP or PB or FITC (53–6.7, BD), CD4 PECy7 (RM4-5, BD), Vβ5.1,5.2 FITC (MR9-4, BD), Vα2 APCCy7 (B20.1, BD), CD44 PE (IM7, BD), CD62L APC (MEL-14, BD), IL-2 PerCP (JES6-5H4), TNF PE (MP6-XT22, BD), IFN-γ APC (XMG1.2, BD), IL-4 PE (11B11, BD), IL-10 PE (JES5-16E3, BD), IL-17 PE (TC11-18H10, BD), CD11a FITC (2D7, BD), CD49d FITC (R1-2, BD), CD38 PE (90, BD), CD27 FITC (LG3A10, BD), CD69 PerCP (H1.2F3, BD), KLRG-1 FITC (2F1, eBioscience), CD25 FITC or PE (7D4 or PC61, BD), CD122 FITC (TM-β1, BD), CD43 PECy7 (1B11, BioLegend), CD45 FITC (30-F11, BD), CD70 PE (FR70, BioLegend), CD71 FITC (C2, BD), CD134 PE (OX-86, BioLegend), CD127 PE (SB/199, BD), CTLA-4 PE (UC10-4B9, eBioscience), CD272 PE (8F4, eBioscience), CD279 FITC (J43, eBioscience), CD274 PE (MIH5, BD), Tim-3 Alexa Fluor 647 (B8.2C12, BioLegend), CD223 PE (C9B7W, BD), CD262 PE (MD5-1, BioLegend), CD254 PE (IK22/5, BioLegend), CD95 PECy7 (Jo2, BD), CD178 PE (MFL3, BD), CD195 FITC (C34-3448, BD), CD197 Alexa Fluor 647 (4B12, BD), CD199 Alexa Fluor 647 (9B1, BioLegend), CD183 PerCP/Cy5.5 (CXCR3-173, BioLegend), CD184 PE (2B11/CXCR4, BD), CXCR7 PE (8F11-M16, BioLegend), β7 PerCP (FIB27, BioLegend), T-bet PE (4B10, BD), Eomes FITC (Dan11mag, eBioscience), GATA-3 PE (L50-823, BD), RORγ-t PE (Q31-378, BD), FOXP3 PE (R16-715, BD), H-2K^b^SIINFEKL PE (25-D1.16, eBioscience), Ki67 e450 (SolA15, eBioscience). To stain biotinylated H-2K^b^-SIINFEKL tetramer was used streptavidin APC or PE (BD). As isotype controls were used IgG1kappa FITC or PE or PECy7 or APC, IgG2a kappa FITC or PE or APC, IgG2b kappa FITC or PE, IgG2 kappa FITC, and IgG1 lambda PE, all from BD.

The transcription of cytokine mRNA in BMDC after *T*. *cruzi* exposure was assessed by RT-PCR. To this end, 10×10^6^ BMDC from each condition were used. Total RNA was extracted with Trizol (Invitrogen) and purified with Quick RNA Miniprep columns (Zymo Research) according to the manufacturer instructions. RNA was quantified in Nanodrop and cDNA was synthesized with SuperScript III kit (Invitrogen), following manufacturer instructions. The absence of genomic DNA was confirmed by using controls to which no reverse transcriptase was added. The resulting cDNA was amplified in a StepOne Plus equipment (Applied Biosystems) with SYBR Green (Thermo Scientific) using specific primers. Relative expression of target genes was normalized using *gapdh* as endogenous control and calculated by the Δ Δ Ct method. Primer sequences are given in S1 Table.

To perform *in vitro* antigen presentation assays, spleen cells from OTI naïve animals were harvested and the CD8^+^ T cell population was isolated through negative selection with CD8^+^ T cell MACS beads isolation kit (Miltenyi) according to the manufacturer specification. The isolated lymphocytes were co-cultured for 5 days with BMDC previously exposed or not to *T*. *cruzi*, stimulated with LPS and loaded with SIINFEKL peptide. The ratio of 1 BMDC to 5 CD8^+^ T cells was used (with 1 x 10^5^ T cells/well) and the number of IFN-γ secreting cells was determined by Elispot as described elsewhere [12,15].

### Statistical analysis

Groups were compared using Two-Way ANOVA followed by Tukey’s HSD test (http://faculty.vassar.edu/lowry/VassarStats.html). Differences were considered significant at a P value of <0.05.

## Supporting Information

S1 Fig Suboptimal expansion and cytokine response of OTI CD8^+^ T cells upon *in vivo* stimulation with *T*. *cruzi*-exposed BMDC-SIINFEKL. OTI cells were adoptively transferred into C57BL/6 mice prior to transfer of control BMDC exposed to LPS only (Gr.1) or BMDC exposed to LPS and loaded with SIINFEKL peptide (Gr. 2) or BMDC previously exposed to *T*. *cruzi* and LPS and loaded with SIINFEKL peptide (Gr. 3). The SIINFEKL-specific immune response was assessed after 5 days. a and b- The numbers of SIINFEKL-specific CD8^+^ T cells were determined by TCR Vα2 Vβ5 staining. c and d- After 5 days, spleen cells were harvested and restimulated *ex vivo* with SIINFEKL peptide. The numbers of IFN-γ-producing CD8^+^ T cells were determined by Elispot (SFC: spot-forming cell). Results are one of three separate experiments expressed as individual values and the mean ± SEM of each group. Asterisks indicate significant differences between groups (****P<0.0001 One-way ANOVA followed by Tukey post-hoc test).
(TIF)

S2 Fig Cytokine response of OTI and parasite-specific CD8^+^ T cells upon *in vivo* stimulation with *T*. *cruzi*-exposed BMDC. OTI cells were adoptively transferred into C57BL/6 mice before the transfer of BMDC, BMDC-SIINFEKL (Gr.2), or *T*. *cruzi-*exposed BMDC-SIINFEKL (Gr.3). After 5 or 12 days, spleen cells were harvested and restimulated *ex vivo* with Medium, SIINFEKL, VNHRFTLV or ANYKFTLV (the last two corresponding to *T*. *cruzi* MHCI-restricted epitopes). TNF and IFN-γ were detected in CD8^+^ T cells by ICS. Plots represent one of four mice for each group.
(TIF)

S3 Fig Unaltered induction of OTI CD8^+^ T cell responses upon immunization with AdASP-2-exposed BMDC-SIINFEKL. a- 1 x 10^4^ OTI cells were adoptively transferred into C57BL/6 mice prior to transfer of 5 x 10^5^ control BMDC exposed to LPS only (Gr.1) or 5 x 10^5^ BMDC exposed to LPS and loaded with SIINFEKL peptide (Gr. 2) or 5 x 10^5^ BMDC previously exposed to AdASP-2 (50 PFU/cell) and LPS and loaded with SIINFEKL peptide (Gr. 3). The SIINFEKL-specific immune response was assessed after 5 days. b- The numbers of SIINFEKL-specific CD8^+^ T cells were determined by TCR Vα2 Vβ5 staining. c—The ability of naïve OTI cells to differentiate into effector cells was evaluated by CD44 and CD62L staining of TCR Vα2 Vβ5 double positive CD8 cells. d- Spleen cells were restimulated *ex vivo* with SIINFEKL peptide and the numbers of TNF and/or IFN-γ-producing CD8^+^ T cells were assessed by ICS. Results are one of two separate experiments expressed as individual values and the mean ± SEM of each group. No differences were found between the indicated groups (One-way ANOVA followed by Tukey post-hoc test).
(TIF)

S4 Fig BMDC exposed to *T*. *cruzi* are able to express cytokine genes and prime CD8^+^ T cells *in vitro*. BMDC were left untreated or exposed to *T*. *cruzi* for 24 h and/or LPS for 6 h. a- Transcription of the indicated cytokines was assessed by RT-PCR. b- After incubation with SIINFEKL peptide, the ability of these cells to prime naïve OTI CD8^+^ T cells *in vitro* was assessed by Elispot to detect IFN-γ after 5 days of co-culture. SFC: spot-forming cell. No difference was detected between the indicated groups (One-way ANOVA followed by Tukey post-hoc test).
(TIF)

S5 Fig Suboptimal expansion and differentiation of OTI CD8^+^ T cells upon *in vivo* stimulation with *T*. *cruzi*-exposed splenic DC co-injected with SIINFEKL-loaded splenic DC. a- Experimental design: 1 x 10^4^ OTI cells were adoptively transferred into C57BL/6 mice before the transfer of 5 x 10^5^ DC (Gr.1), 5 x 10^5^ DC-SIINFEKL (Gr.2) or 5 x 10^5^ DC-SIINFEKL and 5 x 10^5^ *T*. *cruzi*-exposed DC (Gr.3). These DC were isolated with CD11c+ beads from the spleen of C57BL/6 naïve mice. The SIINFEKL-specific immune response was assessed after 5 days. b- Numbers of SIINFEKL-specific CD8^+^ T cells were determined by TCR Vα2 Vβ5 staining. c- The ability of naïve OTI cells to differentiate into effector cells was evaluated by CD44 and CD62L staining of TCR Vα2^+^ Vβ5^+^ CD8^+^ T cells. d- Spleen cells were restimulated *ex vivo* with SIINFEKL peptide and numbers of TNF and/or IFN-γ-producing CD8^+^ T cells were determined by ICS. Results are one of three separate experiments expressed as individual values and the mean ± SEM of each group. Asterisks represent significant differences between the indicated groups (****P<0.0001, One-way ANOVA followed by Tukey post-hoc test).
(TIF)

S6 Fig Phenotype of OTI CD8^+^ T cells upon stimulation with *T*. *cruzi*-exposed BMDC-SIINFEKL. OTI cells were adoptively transferred into C57BL/6 mice before the transfer of BMDC (Gr.1), BMDC-SIINFEKL (Gr.2) or *T*. *cruzi*-exposed BMDC-SIINFEKL (Gr.3). After 5 days, splenic CD8^+^ T cells were stained with H-2K^b^ SIINFEKL tetramers and mAbs to the surface markers and transcription factors indicated. Results are one of three separate experiments expressed as individual values and the mean ± SEM of each group.
(TIF)

S7 Fig CD4^+^ T cells induced by *T*. *cruzi*-exposed BMDC mediate the suppression of OTI CD8^+^ T cell priming. a- *T*. *cruzi*-exposed BMDC were adoptively transferred into C57BL/6 mice and the splenic CD4^+^ T cells were sorted after 5 days. These cells (10 x 10^6^/mouse) were adoptively transferred into C57BL/6 mice on the same day of OTI cell transfer and 24 h before BMDC-SIINFEKL transfer. The SIINFEKL-specific immune response was assessed after 5 days. b and c- The numbers of SIINFEKL-specific CD8^+^ T cells were determined by H-2K^b^-SIINFEKL tetramer staining and TCR Vα2 and Vβ5 staining. d- The ability of naïve OTI cells to differentiate into effector cells was evaluated by CD44 and CD62L staining of TCR Vα2^+^ Vβ5^+^ CD8^+^ T cells. e- Spleen cells were also restimulated *ex vivo* with SIINFEKL peptide and the numbers of TNF and/or IFN-γ-producing CD8^+^ T cells were assessed by ICS. Results are one of two separate experiments expressed as individual values and the mean ± SEM of each group. Asterisks represent significant differences between the indicated groups (**P<0.01, ***P<0.001, ****P<0.0001 One-way ANOVA followed by Tukey post-hoc test).
(TIF)

S8 Fig Suboptimal response of OTI CD8^+^ T cells upon *in vivo* stimulation with *T*. *cruzi*-exposed BMDC-SIINFEKL in *il10* ^*-/-*^ deficient mice. a- OTI cells were adoptively transferred into *il-10* ^-/-^ mice prior to transfer of BMDC (Gr.1), BMDC-SIINFEKL (Gr.2) or *T*. *cruzi*-exposed BMDC-SIINFEKL (Gr.3). The SIINFEKL-specific immune response was assessed after 5 days. b–Phenotype of *il-10* ^-/-^ mice was confirmed by ELISA to quantify IL-10, TNF and IL-6 in the supernatant of BMDC stimulated with LPS. c and d- Numbers of SIINFEKL-specific CD8^+^ T cells were determined by H-2K^b^ SIINFEKL tetramer and TCR Vα2 Vβ5 staining. e- The ability of naïve OTI cells to differentiate into effector cells was evaluated by CD44 and CD62L staining of TCR Vα2^+^ Vβ5^+^ CD8^+^ T cells. f- Spleen cells were restimulated *ex vivo* with SIINFEKL peptide and the numbers of TNF and/or IFN-γ-producing CD8^+^ T cells were assessed by ICS. Results are one of two separate experiments expressed as individual values and the mean ± SEM of each group. Asterisks indicate significant differences between groups (*P<0.05, **P<0.01, ***P<0.001 One-way ANOVA followed by Tukey post-hoc test).
(TIF)

S9 Fig Effect of antibody-mediated CD25^+^ cell depletion in the *in vivo* priming of OTI cells by *T*. *cruzi*-exposed BMDC. a- OTI cells were adoptively transferred into mice prior to transfer of BMDC (Gr.1), BMDC-SIINFEKL (Gr.2) or *T*. *cruzi*-exposed BMDC-SIINFEKL (Gr.3). All animals were treated every 48 h with 0.5 mL of ascite fluid from PC61-injected nude mice. Treatment started one day before OTI cell transfer and followed until the end of the experiment. The SIINFEKL-specific immune response was assessed after 5 days. b- Depletion of CD25^+^ cells was confirmed by staining with 7D4 clone. c- Numbers of SIINFEKL-specific CD8^+^ T cells were determined by H-2K^b^ SIINFEKL tetramer staining. d- The ability of naïve OTI cells to differentiate into effector cells was evaluated by CD44 and CD62L staining of TCR Vα2^+^ Vβ5^+^ CD8^+^ T cells. e- Spleen cells were restimulated *ex vivo* with SIINFEKL peptide and the numbers of TNF and/or IFN-γ-producing CD8^+^ T cells were assessed by ICS. Results are one of two separate experiments expressed as individual values and the mean ± SEM of each group. Asterisks indicate significant differences between groups (*P<0.05, **P<0.01, ***P<0.001 One-way ANOVA followed by Tukey post-hoc test).
(TIF)

S1 Table Primers used in RT-PCR for detecting mRNA levels of cytokines in BMDC. (TIF)
