# Correction: A Human Trypanosome Suppresses CD8^+^ T Cell Priming by Dendritic Cells through the Induction of Immune Regulatory CD4^+^ Foxp3^+^ T Cells

**DOI:** 10.1371/journal.ppat.1005771

**Published:** 2016-07-11

**Authors:** Jonatan Ersching, Alexandre Salgado Basso, Vera Lúcia Garcia Calich, Karina Ramalho Bortoluci, Maurício M. Rodrigues

The third author's name is spelled incorrectly. The correct name is: Vera Lúcia Garcia Calich. The correct citation is: Ersching J, Basso AS, Calich VLG, Bortoluci KR, Rodrigues MM (2016) A Human Trypanosome Suppresses CD8+ T Cell Priming by Dendritic Cells through the Induction of Immune Regulatory CD4+ Foxp3+ T Cells. PLoS Pathog 12(6): e1005698. doi:10.1371/journal.ppat.1005698
