# Supplementary figures and images for: A Human Trypanosome Suppresses CD8+ T Cell Priming by Dendritic Cells through the Induction of Immune Regulatory CD4+ Foxp3+ T Cells
Source: PLoS Pathog. 2016 Jun 22;12(6):e1005698. doi: 10.1371/journal.ppat.1005698 (PMC4917094; doi:10.1371/journal.ppat.1005698)

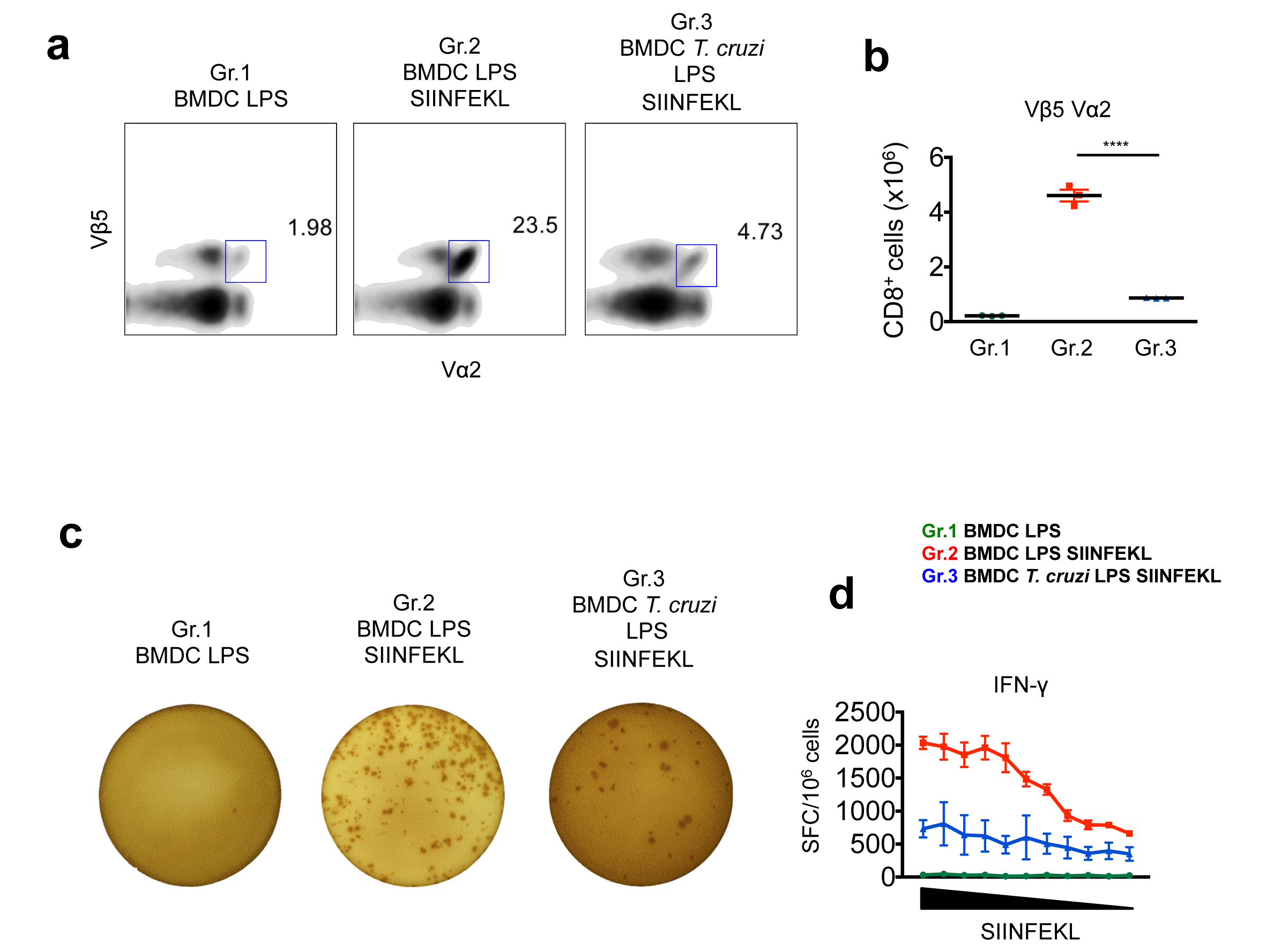

Supplement: S1 Fig — OTI cells were adoptively transferred into C57BL/6 mice prior to transfer of control BMDC exposed to LPS only (Gr.1) or BMDC exposed to LPS and loaded with SIINFEKL peptide (Gr. 2) or BMDC previously exposed to T. cruzi and LPS and loaded with SIINFEKL peptide (Gr. 3). The SIINFEKL-specific immune response was assessed after 5 days. a and b- The numbers of SIINFEKL-specific CD8+ T cells were determined by TCR Vα2 Vβ5 staining. c and d- After 5 days, spleen cells were harvested and restimulated ex vivo with SIINFEKL peptide. The numbers of IFN-γ-producing CD8+ T cells were determined by Elispot (SFC: spot-forming cell). Results are one of three separate experiments expressed as individual values and the mean ± SEM of each group. Asterisks indicate significant differences between groups (****P<0.0001 One-way ANOVA followed by Tukey post-hoc test). (TIF) [file ppat.1005698.s001.tif]

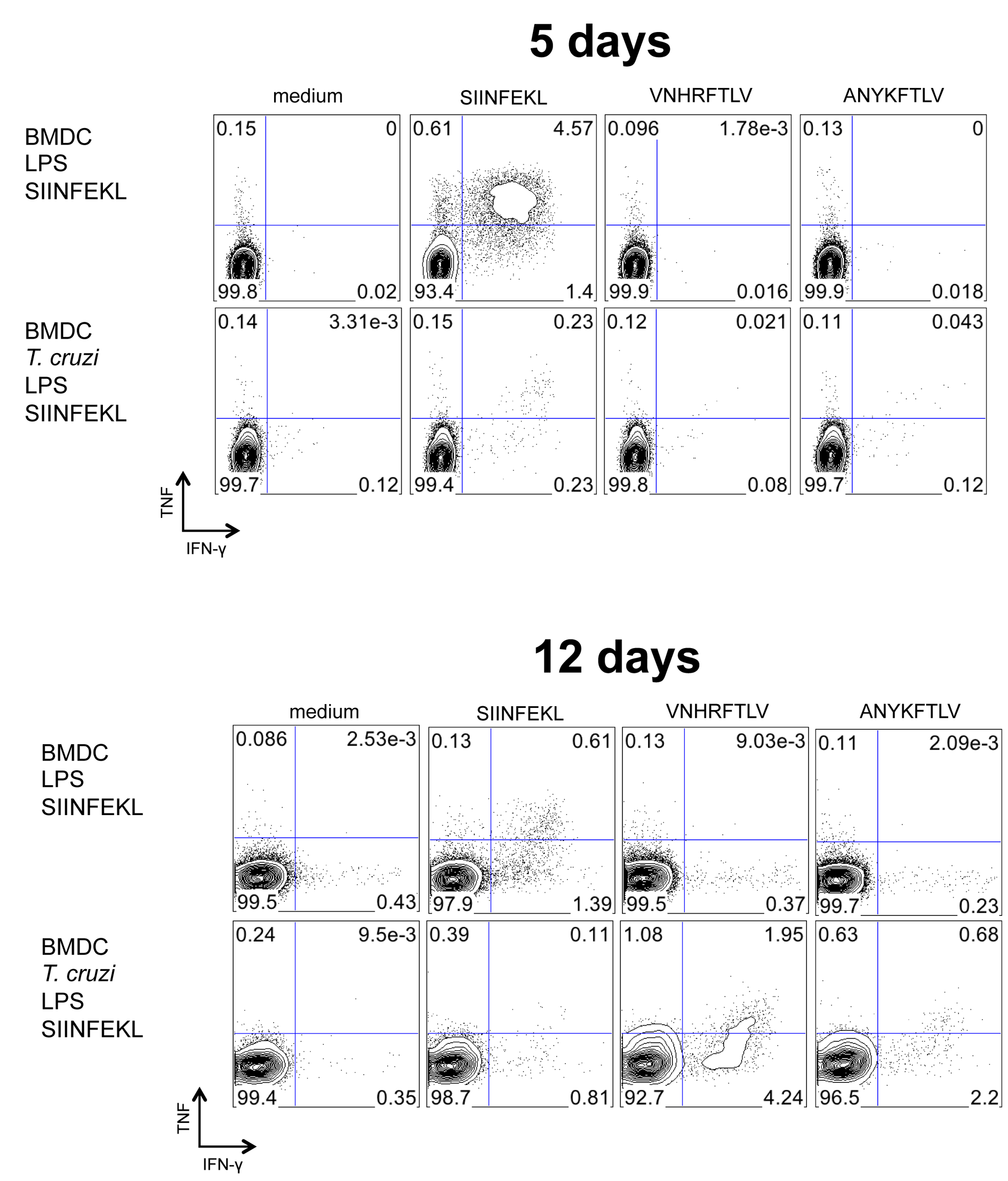

Supplement: S2 Fig — OTI cells were adoptively transferred into C57BL/6 mice before the transfer of BMDC, BMDC-SIINFEKL (Gr.2), or T. cruzi-exposed BMDC-SIINFEKL (Gr.3). After 5 or 12 days, spleen cells were harvested and restimulated ex vivo with Medium, SIINFEKL, VNHRFTLV or ANYKFTLV (the last two corresponding to T. cruzi MHCI-restricted epitopes). TNF and IFN-γ were detected in CD8+ T cells by ICS. Plots represent one of four mice for each group. (TIF) [file ppat.1005698.s002.tif]

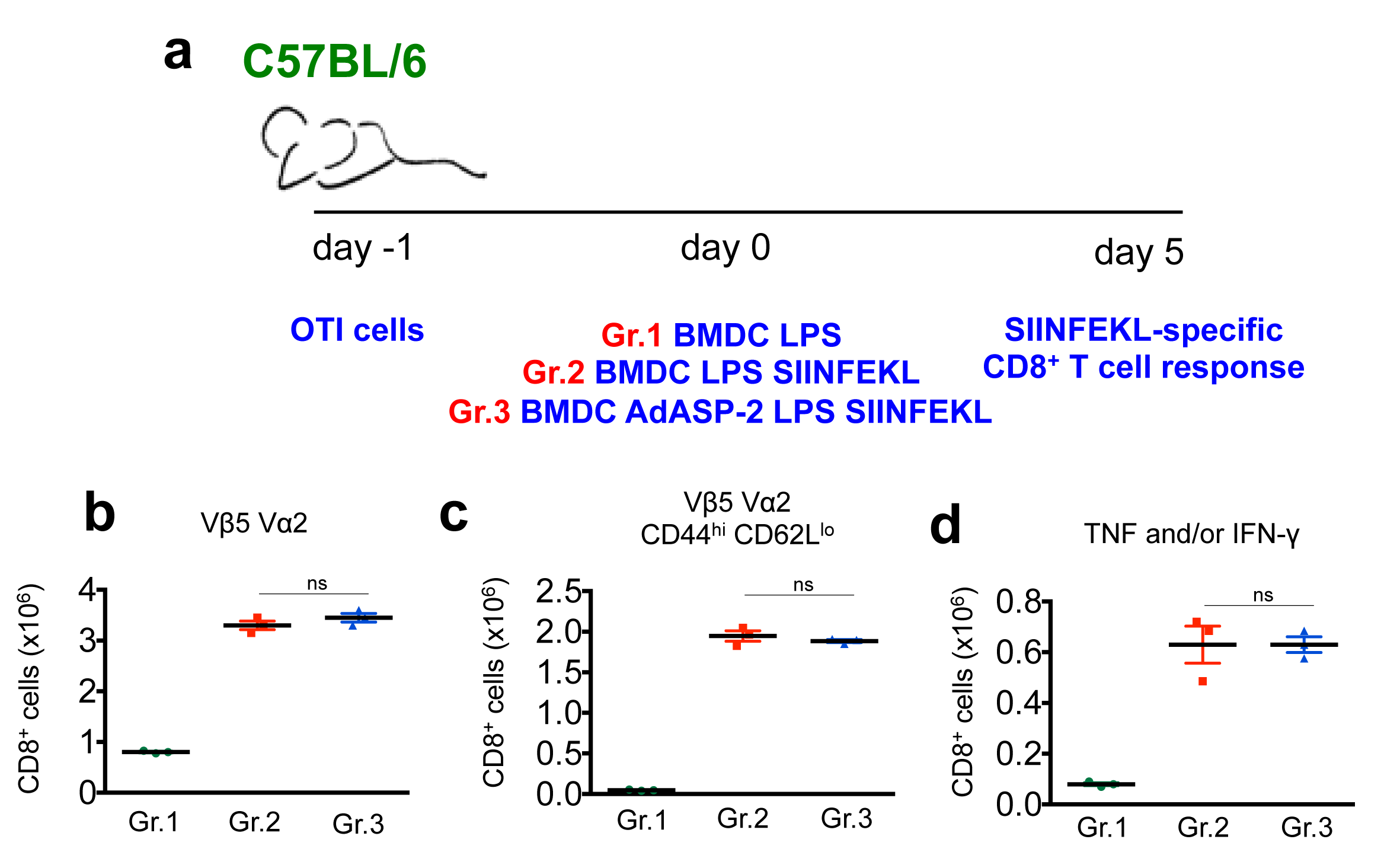

Supplement: S3 Fig — a- 1 x 10^4^ OTI cells were adoptively transferred into C57BL/6 mice prior to transfer of 5 x 10^5^ control BMDC exposed to LPS only (Gr.1) or 5 x 10^5^ BMDC exposed to LPS and loaded with SIINFEKL peptide (Gr. 2) or 5 x 10^5^ BMDC previously exposed to AdASP-2 (50 PFU/cell) and LPS and loaded with SIINFEKL peptide (Gr. 3). The SIINFEKL-specific immune response was assessed after 5 days. b- The numbers of SIINFEKL-specific CD8+ T cells were determined by TCR Vα2 Vβ5 staining. c—The ability of naïve OTI cells to differentiate into effector cells was evaluated by CD44 and CD62L staining of TCR Vα2 Vβ5 double positive CD8 cells. d- Spleen cells were restimulated ex vivo with SIINFEKL peptide and the numbers of TNF and/or IFN-γ-producing CD8+ T cells were assessed by ICS. Results are one of two separate experiments expressed as individual values and the mean ± SEM of each group. No differences were found between the indicated groups (One-way ANOVA followed by Tukey post-hoc test). (TIF) [file ppat.1005698.s003.tif]

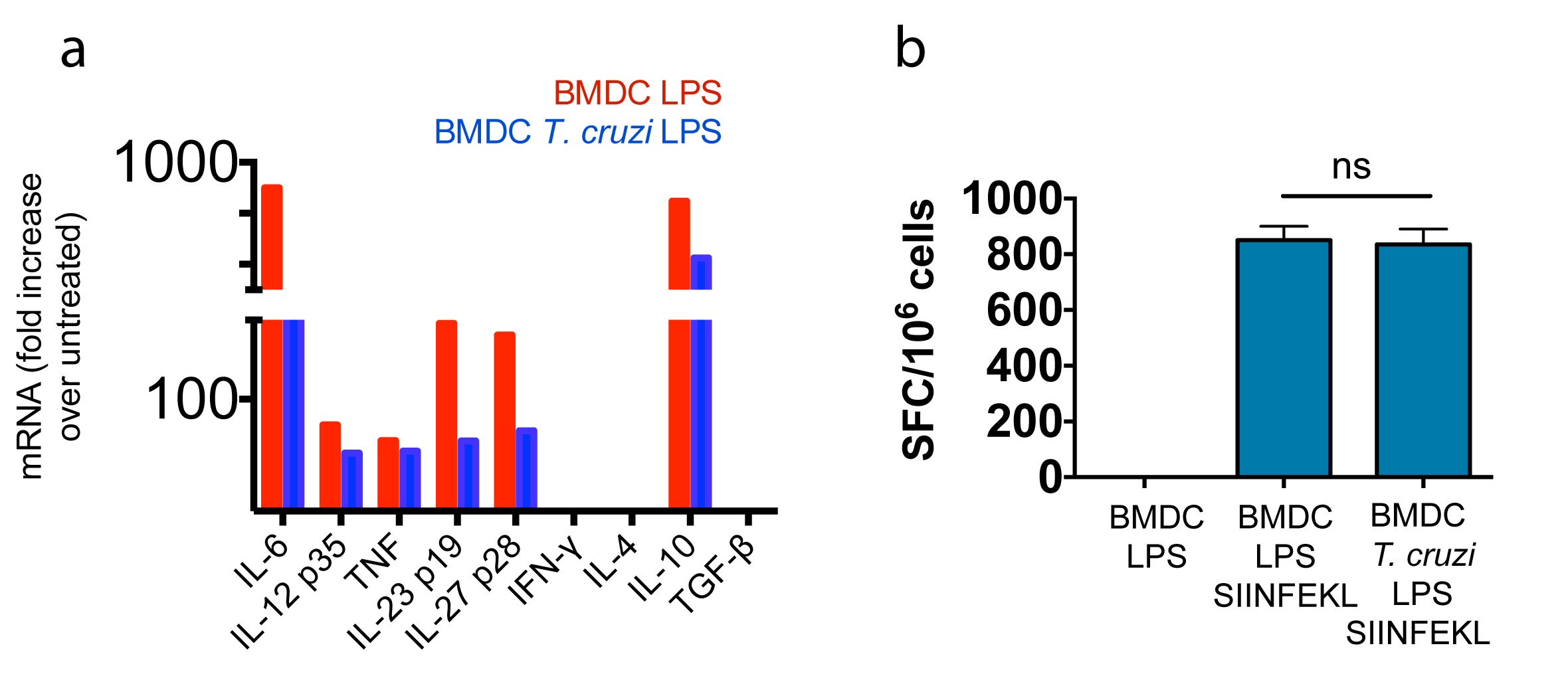

Supplement: S4 Fig — BMDC were left untreated or exposed to T. cruzi for 24 h and/or LPS for 6 h. a- Transcription of the indicated cytokines was assessed by RT-PCR. b- After incubation with SIINFEKL peptide, the ability of these cells to prime naïve OTI CD8+ T cells in vitro was assessed by Elispot to detect IFN-γ after 5 days of co-culture. SFC: spot-forming cell. No difference was detected between the indicated groups (One-way ANOVA followed by Tukey post-hoc test). (TIF) [file ppat.1005698.s004.tif]

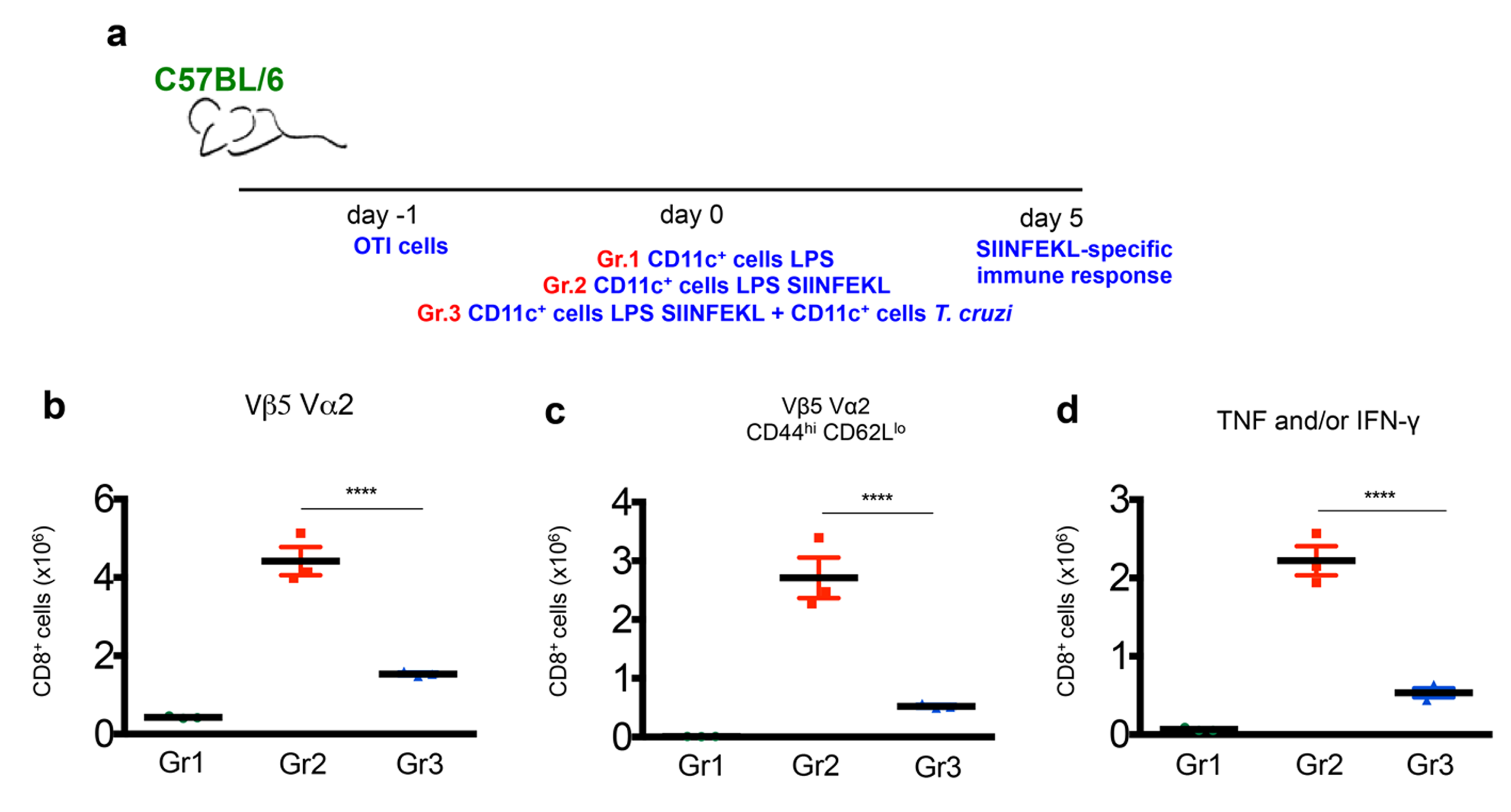

Supplement: S5 Fig — a- Experimental design: 1 x 10^4^ OTI cells were adoptively transferred into C57BL/6 mice before the transfer of 5 x 10^5^ DC (Gr.1), 5 x 10^5^ DC-SIINFEKL (Gr.2) or 5 x 10^5^ DC-SIINFEKL and 5 x 10^5^ T. cruzi-exposed DC (Gr.3). These DC were isolated with CD11c+ beads from the spleen of C57BL/6 naïve mice. The SIINFEKL-specific immune response was assessed after 5 days. b- Numbers of SIINFEKL-specific CD8+ T cells were determined by TCR Vα2 Vβ5 staining. c- The ability of naïve OTI cells to differentiate into effector cells was evaluated by CD44 and CD62L staining of TCR Vα2+ Vβ5+ CD8+ T cells. d- Spleen cells were restimulated ex vivo with SIINFEKL peptide and numbers of TNF and/or IFN-γ-producing CD8+ T cells were determined by ICS. Results are one of three separate experiments expressed as individual values and the mean ± SEM of each group. Asterisks represent significant differences between the indicated groups (****P<0.0001, One-way ANOVA followed by Tukey post-hoc test). (TIF) [file ppat.1005698.s005.tif]

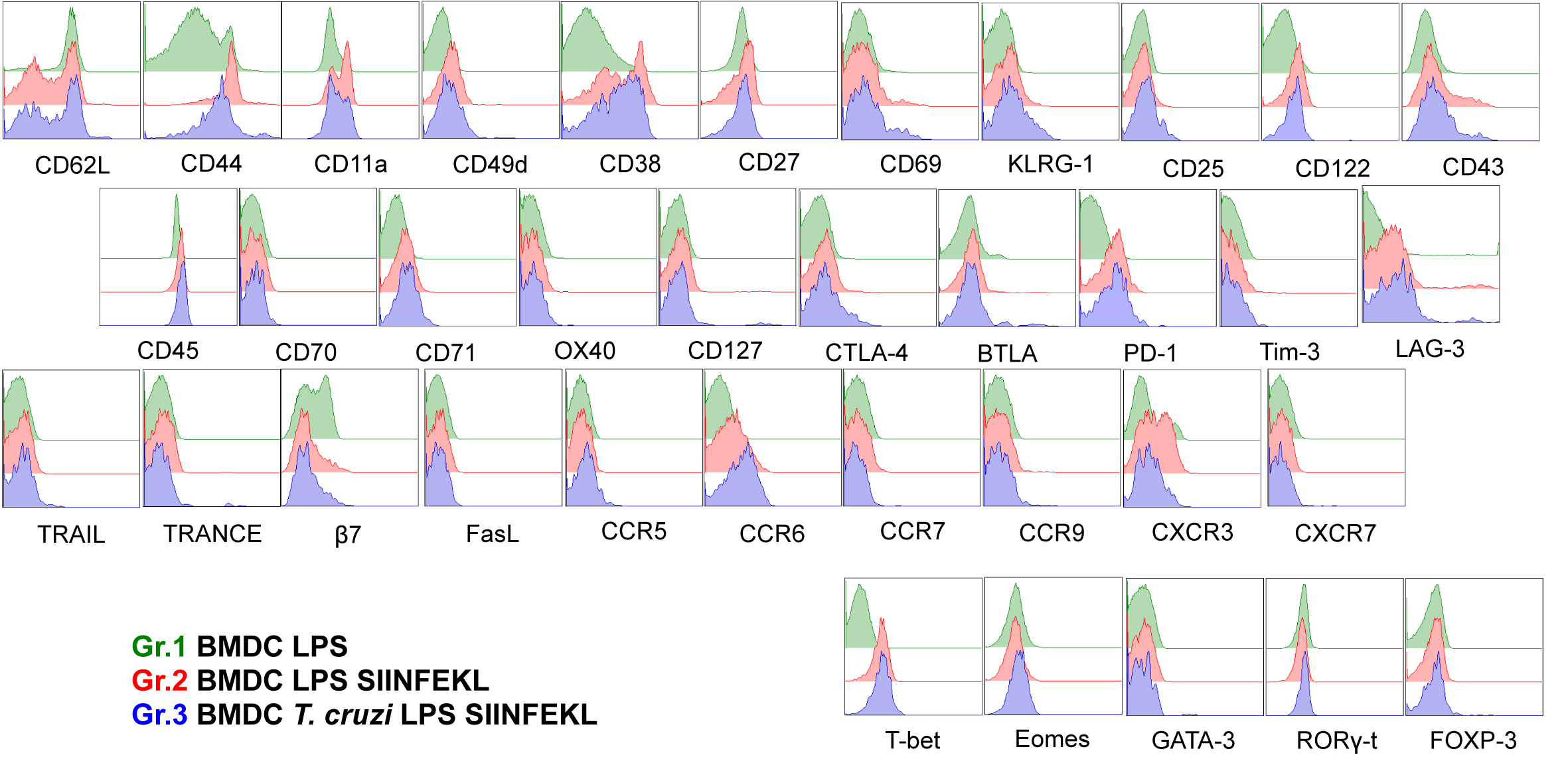

Supplement: S6 Fig — OTI cells were adoptively transferred into C57BL/6 mice before the transfer of BMDC (Gr.1), BMDC-SIINFEKL (Gr.2) or T. cruzi-exposed BMDC-SIINFEKL (Gr.3). After 5 days, splenic CD8+ T cells were stained with H-2Kb SIINFEKL tetramers and mAbs to the surface markers and transcription factors indicated. Results are one of three separate experiments expressed as individual values and the mean ± SEM of each group. (TIF) [file ppat.1005698.s006.tif]

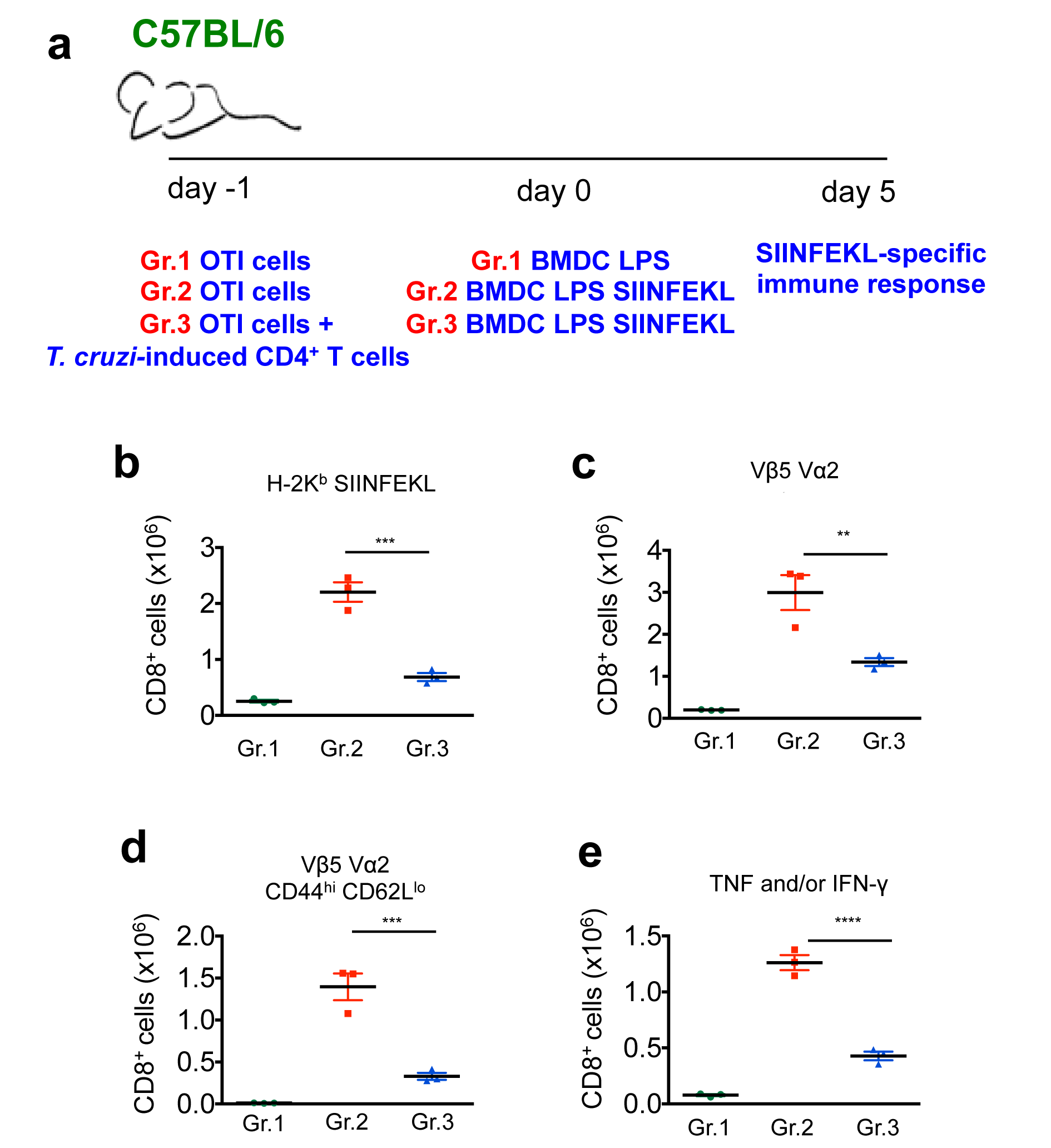

Supplement: S7 Fig — a- T. cruzi-exposed BMDC were adoptively transferred into C57BL/6 mice and the splenic CD4+ T cells were sorted after 5 days. These cells (10 x 106/mouse) were adoptively transferred into C57BL/6 mice on the same day of OTI cell transfer and 24 h before BMDC-SIINFEKL transfer. The SIINFEKL-specific immune response was assessed after 5 days. b and c- The numbers of SIINFEKL-specific CD8+ T cells were determined by H-2Kb-SIINFEKL tetramer staining and TCR Vα2 and Vβ5 staining. d- The ability of naïve OTI cells to differentiate into effector cells was evaluated by CD44 and CD62L staining of TCR Vα2+ Vβ5+ CD8+ T cells. e- Spleen cells were also restimulated ex vivo with SIINFEKL peptide and the numbers of TNF and/or IFN-γ-producing CD8+ T cells were assessed by ICS. Results are one of two separate experiments expressed as individual values and the mean ± SEM of each group. Asterisks represent significant differences between the indicated groups (**P<0.01, ***P<0.001, ****P<0.0001 One-way ANOVA followed by Tukey post-hoc test). (TIF) [file ppat.1005698.s007.tif]

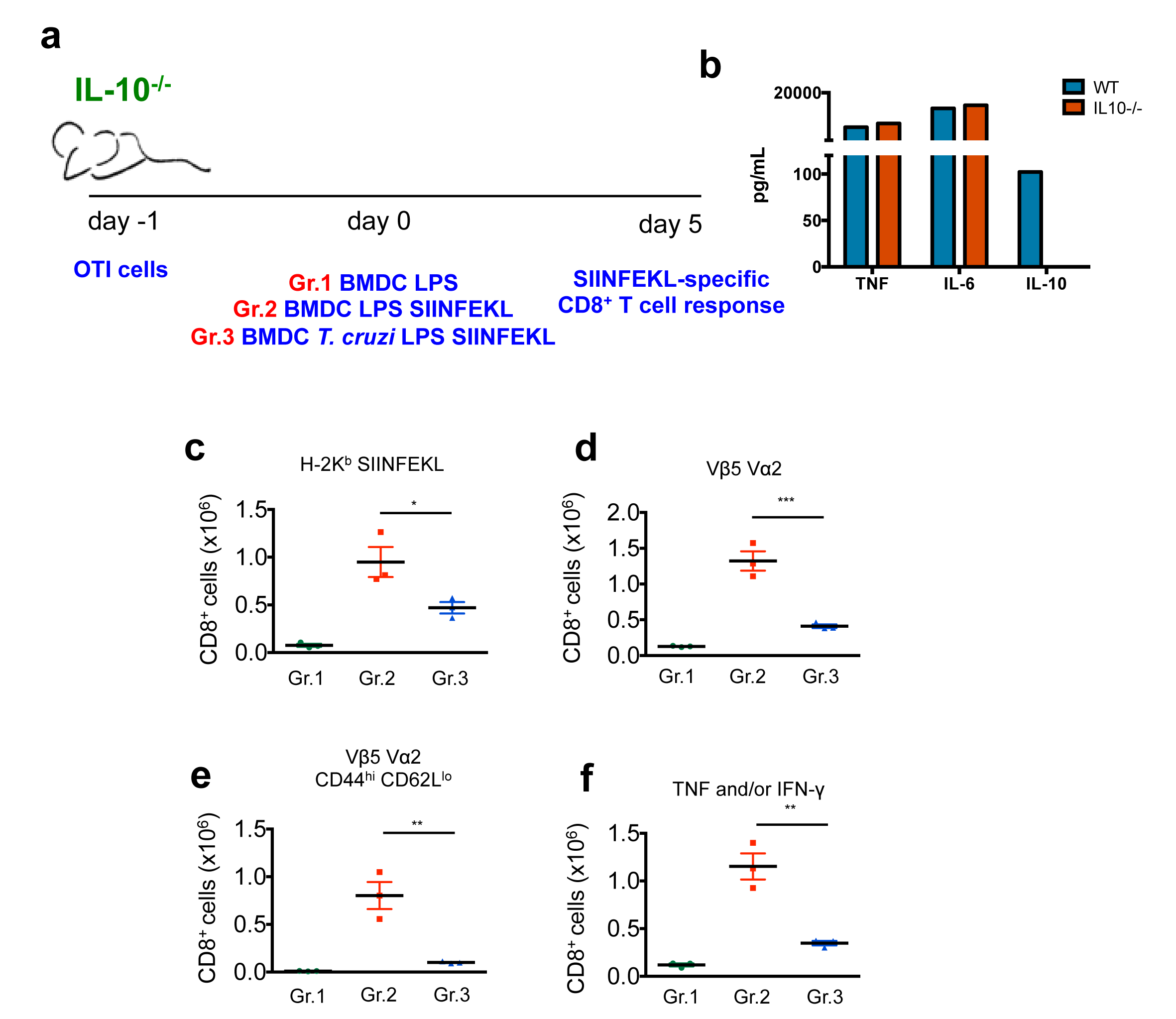

Supplement: S8 Fig — a- OTI cells were adoptively transferred into il-10 -/- mice prior to transfer of BMDC (Gr.1), BMDC-SIINFEKL (Gr.2) or T. cruzi-exposed BMDC-SIINFEKL (Gr.3). The SIINFEKL-specific immune response was assessed after 5 days. b–Phenotype of il-10 -/- mice was confirmed by ELISA to quantify IL-10, TNF and IL-6 in the supernatant of BMDC stimulated with LPS. c and d- Numbers of SIINFEKL-specific CD8+ T cells were determined by H-2Kb SIINFEKL tetramer and TCR Vα2 Vβ5 staining. e- The ability of naïve OTI cells to differentiate into effector cells was evaluated by CD44 and CD62L staining of TCR Vα2+ Vβ5+ CD8+ T cells. f- Spleen cells were restimulated ex vivo with SIINFEKL peptide and the numbers of TNF and/or IFN-γ-producing CD8+ T cells were assessed by ICS. Results are one of two separate experiments expressed as individual values and the mean ± SEM of each group. Asterisks indicate significant differences between groups (*P<0.05, **P<0.01, ***P<0.001 One-way ANOVA followed by Tukey post-hoc test). (TIF) [file ppat.1005698.s008.tif]

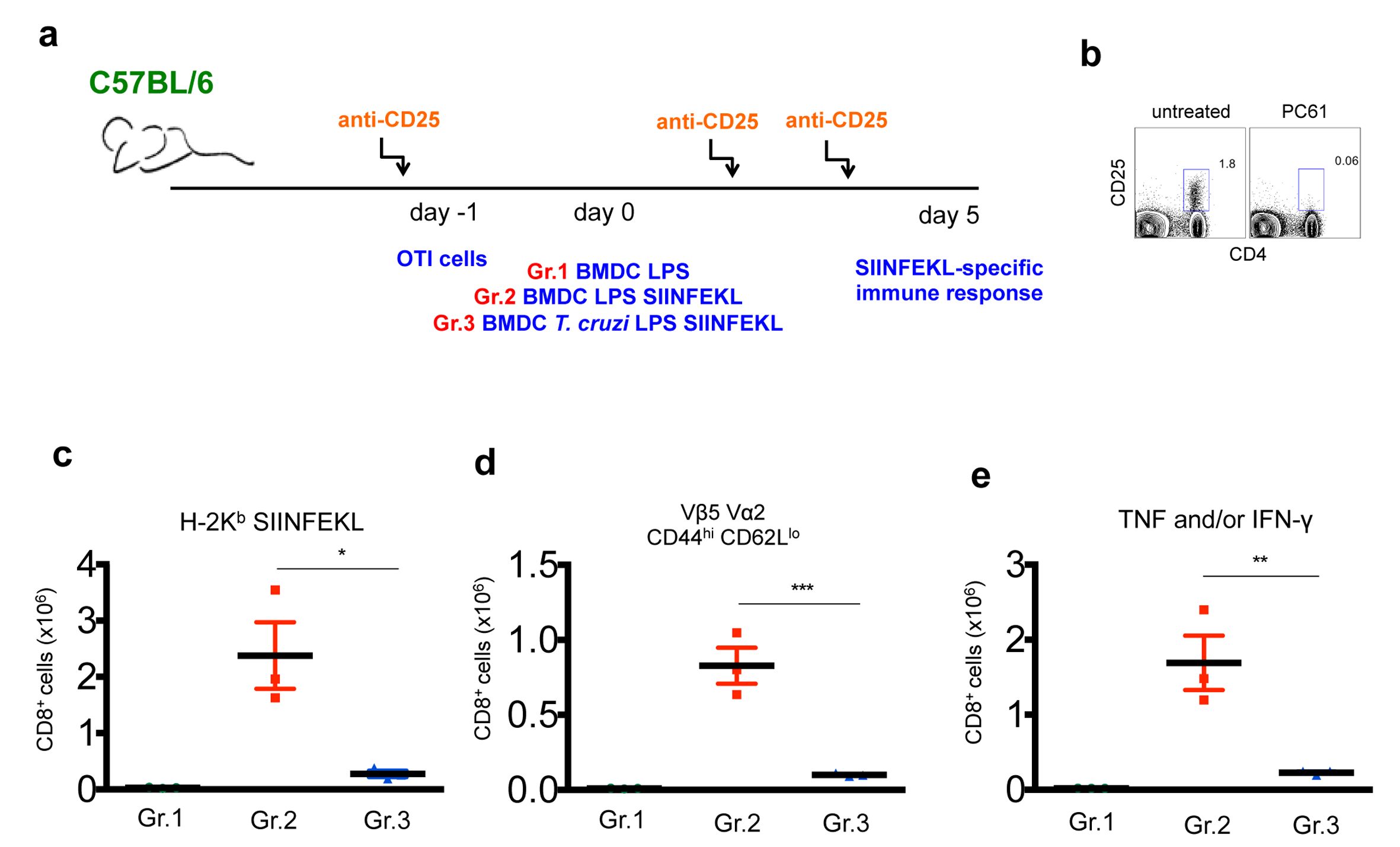

Supplement: S9 Fig — a- OTI cells were adoptively transferred into mice prior to transfer of BMDC (Gr.1), BMDC-SIINFEKL (Gr.2) or T. cruzi-exposed BMDC-SIINFEKL (Gr.3). All animals were treated every 48 h with 0.5 mL of ascite fluid from PC61-injected nude mice. Treatment started one day before OTI cell transfer and followed until the end of the experiment. The SIINFEKL-specific immune response was assessed after 5 days. b- Depletion of CD25+ cells was confirmed by staining with 7D4 clone. c- Numbers of SIINFEKL-specific CD8+ T cells were determined by H-2Kb SIINFEKL tetramer staining. d- The ability of naïve OTI cells to differentiate into effector cells was evaluated by CD44 and CD62L staining of TCR Vα2+ Vβ5+ CD8+ T cells. e- Spleen cells were restimulated ex vivo with SIINFEKL peptide and the numbers of TNF and/or IFN-γ-producing CD8+ T cells were assessed by ICS. Results are one of two separate experiments expressed as individual values and the mean ± SEM of each group. Asterisks indicate significant differences between groups (*P<0.05, **P<0.01, ***P<0.001 One-way ANOVA followed by Tukey post-hoc test). (TIF) [file ppat.1005698.s009.tif]

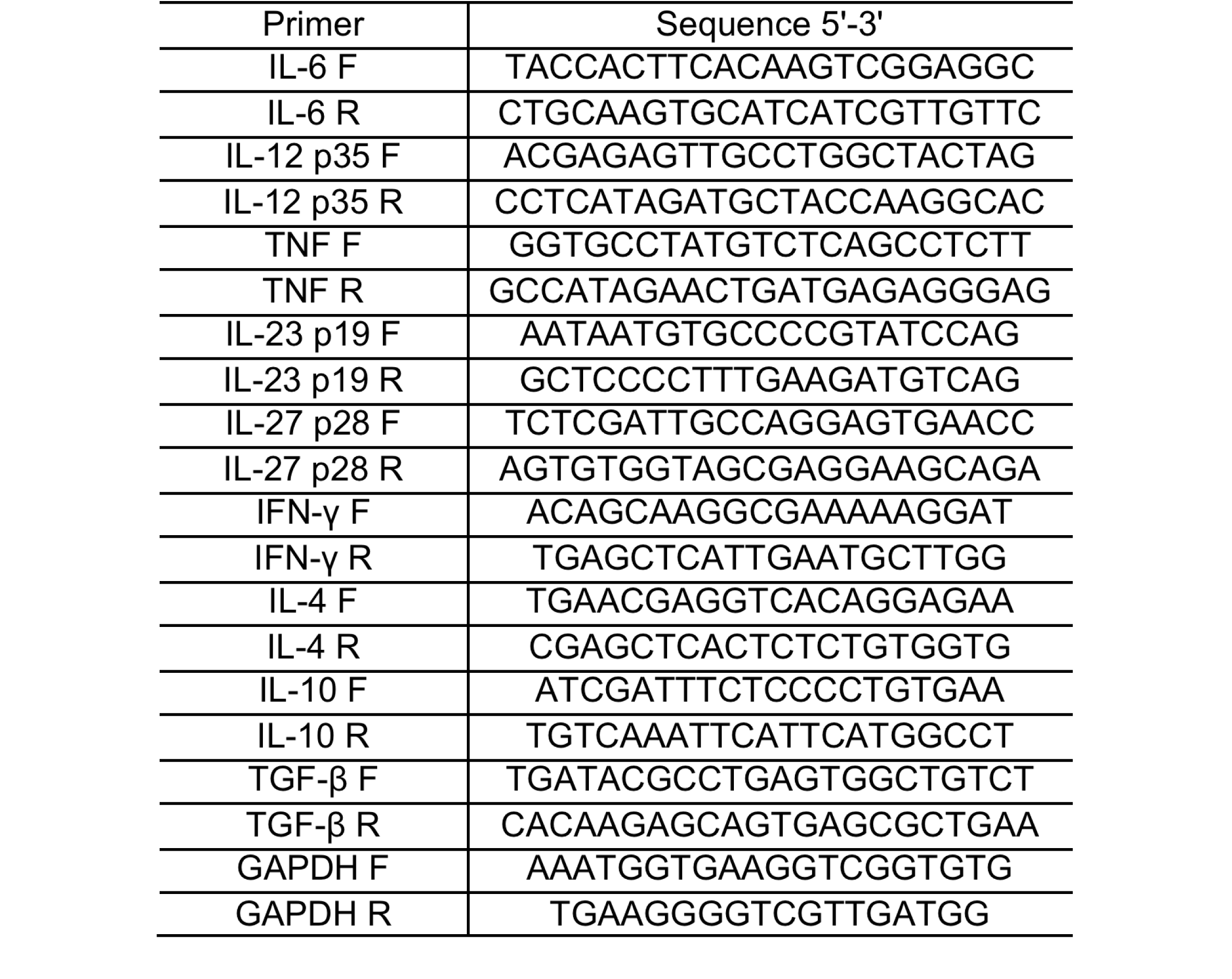

Supplement: S1 Table — (TIF) [file ppat.1005698.s010.tif]
